# Experimental Characterization and Numerical Assessment of the LONGASF Asphalt-Supported Ballastless Railway Track System

**DOI:** 10.3390/ma19204415

**Published:** 2026-10-20

**Authors:** Łukasz Chudyba, Małgorzata Urbanek, Dariusz Kudła

**Affiliations:** Faculty of Civil Engineering, Cracow University of Technology, 31-155 Kraków, Poland; malgorzata.urbanek@pk.edu.pl (M.U.); dariusz.kudla@pk.edu.pl (D.K.)

**Keywords:** ballastless track, asphalt supporting layer, longitudinal concrete sleeper, rail fastening system, experimental characterization, finite element analysis, track dynamics, foundation stiffness

## Abstract

The LONGASF ballastless track system combines longitudinal WZA concrete sleepers, an asphalt supporting layer, and the ICOSTRUN-03 fastening system. Although selected elements of the concept and preliminary numerical models have been reported previously, this paper presents, for the first time, the experimental characterization of the fastening system and an integrated assessment of the track response under vertical, transverse, and longitudinal loading. Laboratory tests were conducted to determine vertical stiffness, longitudinal and transverse rail resistance, and performance under repeated loading, including dynamic rail and sleeper displacement measurements. The experimentally identified parameters were subsequently implemented in simplified dynamic models and three-dimensional finite element models. After repeated loading, changes in longitudinal rail resistance and vertical stiffness remained within the limits adopted in the experimental programme. The numerical analyses demonstrated a strong dependence of track response on foundation stiffness and model extent. In the extended three-dimensional model, the maximum calculated horizontal and vertical displacements were 2.439 and 2.434 mm, respectively, for the weakest foundation condition considered. The simplified dynamic model indicated that sleeper acceleration may exceed the adopted 1 g criterion at the highest analysed speeds. However, because the model did not include vehicle inertia or coupled wheel–rail interaction, 250 km/h should be regarded solely as a parametric calculation case rather than a validated operating speed. The results provide a preliminary numerical assessment of the LONGASF concept and identify asphalt temperature sensitivity, foundation stiffness, fastening-system optimization, and full-scale model validation as key areas for further research.

## 1. Introduction

The development and increasing implementation of unconventional railway track systems are closely related to the need to reduce railway infrastructure maintenance costs, as well as to the influence of technological progress and investment financing policies. This raises the question of whether conventional ballasted track systems remain the most rational solution and whether currently available unconventional track structures effectively reduce both construction and life-cycle maintenance costs. Unconventional railway track systems are generally defined as structures that, unlike traditional track forms, do not contain a ballast layer. Instead, the supporting structure typically consists of several layers made of hydraulically stabilized soil, asphalt mixtures, soil–cement composites, or concrete elements. The supporting base may also take the form of prefabricated or cast-in-place concrete slabs, as well as engineering structures such as bridges and tunnels. Such solutions have been used for many years, initially on a limited scale, primarily in tunnels, on bridges, within transition zones adjacent to engineering structures where gradual stiffness variation was required, and in station areas exposed to accelerated ballast degradation. Their application in tunnels and on bridges additionally enabled a reduction in the overall structural height of the track system. Ballastless track systems (slab tracks) currently represent one of the principal directions in railway infrastructure development, particularly in high-speed rail networks. Numerous studies have highlighted their advantages over conventional ballasted tracks, including superior geometric stability, improved operational performance, and reduced maintenance requirements. However, these benefits are accompanied by increased sensitivity to thermal actions and construction inaccuracies [1,2,3,4,5,6,7,8]. The development of slab track systems has followed an evolutionary path and has varied considerably across different regions of the world, from European solutions to rapidly expanding applications in Asia. Significant progress has been achieved in China through the implementation of High-Speed Railway systems, while in Europe, development has been strongly influenced by German and Slovak experiences, including investigations of unconventional slab track configurations [4,5,6,9]. Early design studies and international guidelines emphasized the importance of appropriate layer composition and the use of optimized substructure systems, including unbound foundation layers [10,11,12]. Among contemporary ballastless track systems, the CRTS III structure is regarded as one of the most advanced solutions. It is characterized by a multilayer configuration in which the interfaces between individual structural layers play a critical role. Experimental investigations have demonstrated that the mechanical performance of track slabs is highly dependent on both material properties and construction technology [13,14,15,16]. Numerical and probabilistic analyses have further shown that thermal loading significantly affects the stress state of the structure and may lead to the development of self-induced stresses and the initiation of degradation processes [14]. Moreover, environmental conditions, including climatic factors and railway line location, directly influence temperature distributions within the slab, as confirmed by previous studies [17]. One of the most important operational challenges associated with ballastless tracks is the progressive degradation of interlayer interfaces resulting from the combined effects of mechanical and thermal loading. Field investigations have demonstrated that both passenger and freight traffic contribute to the accumulation of fatigue damage and the deterioration of contact properties between structural layers [18,19]. Furthermore, specific geotechnical conditions, such as tunnel floor heave and subgrade deformations, may significantly influence damage development and stress redistribution within the track structure [18,20]. These phenomena are particularly pronounced in tunnels, where restricted structural deformations create unique loading conditions [10], and in specialized track slab systems designed to accommodate both railway and road vehicles [21]. Degradation processes are further intensified when ballastless tracks are installed on engineering structures. In bridge applications, increased susceptibility to damage at layer interfaces has been observed due to dynamic loading effects and deformations of the supporting structure [11,22]. The track–bridge interaction problem remains a complex engineering issue, as differences in stiffness and imposed displacements generate additional stresses within the track system [23]. Similar aspects have also been investigated in studies concerning rail behaviour under compressive forces in ballastless track systems, which is of particular importance for overall track stability [24]. To improve the understanding of degradation mechanisms, advanced numerical modelling approaches have been developed to investigate the behaviour of multilayer ballastless track systems. These methods enable the analysis of interface performance, cyclic loading effects, and nonlinear material behaviour, providing valuable insight into the long-term durability of track structures [15,16]. However, their practical application often requires extensive calibration using experimental data, which remains a significant limitation. Review studies indicate that ballastless track degradation is a multifactorial process involving material deterioration, geometric changes, and functional performance loss [25,26]. Another important research direction concerns alternative structural and material solutions. The incorporation of asphalt layers and bituminous mixtures into railway track structures has been investigated as a means of improving vibration damping characteristics and reducing stress concentrations [27]. However, limited information is available on systems combining longitudinal concrete sleepers with an asphalt supporting layer and a dedicated fastening system. In particular, the literature lacks integrated studies in which experimentally determined fastening characteristics are directly implemented in numerical models covering vertical, transverse, and longitudinal track response. The influence of foundation stiffness and adjacent fastening systems on the predicted behaviour of such structures also remains insufficiently documented. Furthermore, the temperature-dependent stiffness of asphalt should be considered when interpreting numerical results.

Selected elements of the LONGASF concept and preliminary numerical models were reported previously [28,29]. However, the experimental characterization of selected mechanical properties of the ICOSTRUN-03 fastening system and the integrated assessment of track response under vertical, transverse, and longitudinal loading are presented publicly here for the first time. The study combines experimentally identified fastening characteristics, simplified dynamic analyses over a speed range of 0–250 km/h, and three-dimensional numerical analyses accounting for the influence of adjacent fastening systems. The main contribution is the first comprehensive evaluation of these previously unpublished project results, their cross-comparison with the published model components, and an explicit identification of the validation steps required before practical implementation.

## 2. Ballastless Track Systems

To achieve the required stiffness and vibration-damping performance, ballastless track structures employ materials with carefully controlled mechanical properties capable of maintaining their performance under significant dynamic loading. The design of multilayer ballastless track systems is based on the selection of appropriate material properties and layer thicknesses to ensure that the structure operates predominantly within the elastic range. A fundamental design principle adopted for ballastless track systems is the gradual reduction in stiffness from the rail towards the subgrade. Consequently, the rail, fastening system, sleeper, supporting layers, frost protection layer, and subgrade form a multilayer structure with progressively decreasing stiffness. The design methodology is largely derived from approaches used in pavement engineering, where parameters such as the California Bearing Ratio (CBR), subgrade elastic modulus, and deformation modulus are commonly employed.

Depending on the adopted structural concept, the rail may be supported continuously by a slab or discretely by sleepers, blocks, or fastening points. The supporting structure may consist of cast-in-place concrete, prefabricated concrete elements, asphalt concrete, hydraulically bound layers, or combinations of these materials. Its geometry and mechanical properties are determined to ensure adequate load transfer and protection of the subgrade. Simplified design approaches may assume load spreading through successive layers at an angle of approximately 45° [30]; however, detailed assessment requires consideration of the actual stiffness and thickness of each layer. Ballastless track systems may therefore be broadly classified as structures with continuous rail support and structures with discrete rail support. Representative solutions are summarized below [4,5,6,31,32]:

- Concrete systems with direct fastening to the slab or with bi-block sleepers embedded within the slab (e.g., RHEDA 2000, RHEDA BERLIN), as well as systems with transversely prestressed prefabricated slabs (e.g., Bögl, Porr Slab Track, Arianna Slab Track, Shinkansen Slab Track);
- Channel slab systems (e.g., Edilon ERS or similar), featuring rail channels integrated into reinforced concrete or composite slabs, as well as systems employing steel rail channels;
- Systems with discrete support blocks (e.g., LVT—TRIBETON (Vigirail), EBS—Edilon);
- Systems with direct fastening to a concrete slab (Vossloh System 336);
- Systems utilizing asphalt concrete as a supporting layer for the track superstructure (e.g., GETRAC, SATO, among others);
- Systems with direct support on bridge deck beams.

**Figure 1:**
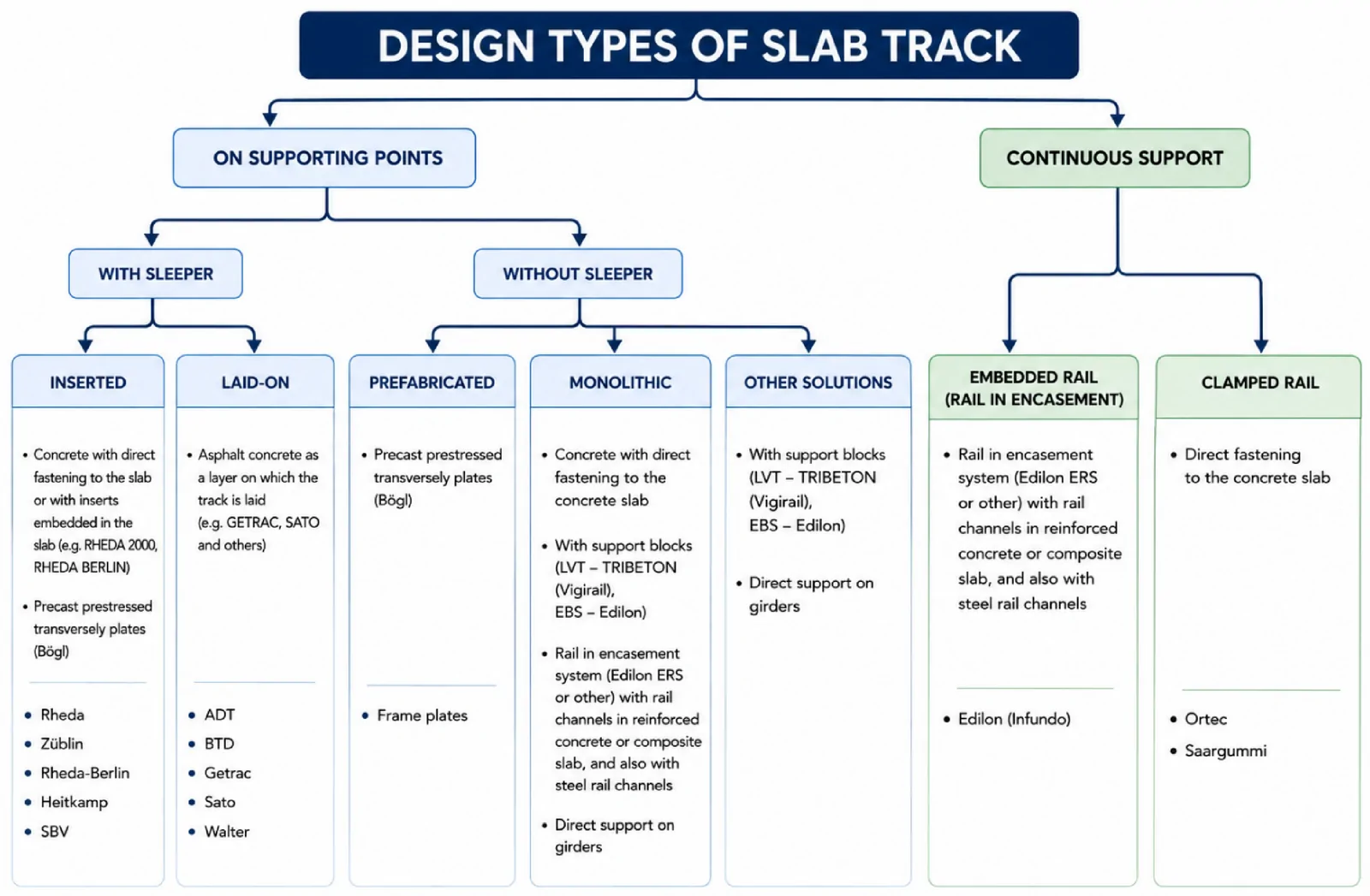
Representative structural configurations of ballastless track systems.

Figure 1 presents a schematic classification of the principal ballastless track system types.

Ballastless track systems may be broadly classified according to the method of rail support and the material of the supporting structure, as summarized schematically in Figure 1. Monolithic concrete systems include solutions with sleepers or support blocks embedded in a reinforced-concrete slab, such as the RHEDA 2000 system [33]. Systems based on prefabricated concrete slabs include the Max Bögl system [34] and the PORR slab-track solution [35]. In embedded rail systems, the rail is continuously supported within a channel filled with an elastic material; the Corkelast ERS solution [36] is an example of this category. Other systems provide discrete rail support directly on a concrete slab or on individual concrete blocks. Asphalt-supported solutions use an asphalt concrete layer as part of the load-bearing structure; the GETRAC [37] system is a representative example. Ballastless configurations may also be installed directly on bridge decks or other engineering structures, subject to appropriate provisions for track–structure interaction.

Experience gained from the implementation of ballastless track systems, particularly in German railway infrastructure, has led to the development of specific design and construction requirements [30,33,35,36,37]. Particular attention is given to the quality and bearing capacity of supporting layers, subgrade preparation, transition zones, construction tolerances, and vibration-mitigation measures. In asphalt-supported systems, the load-bearing structure typically consists of asphalt concrete placed on a hydraulically bound layer and a properly prepared subgrade. The individual layers must provide adequate load distribution, long-term durability, and protection against environmental effects.

Owing to their relatively high stiffness, ballastless track systems may generate greater vibroacoustic effects than conventional ballasted tracks. Resilient rail pads, elastic mats, under-sleeper elements, and floating-slab systems are therefore used to limit vibration transmission and improve passenger comfort. The principal advantages of ballastless track systems include high geometric stability, reduced maintenance requirements, and reduced structural height in tunnels and on engineering structures. These benefits are accompanied by stringent construction tolerances, demanding subgrade requirements, and more complex repair procedures.

Existing ballastless track technologies also have practical limitations. Advanced fastening systems are frequently protected by patents, while their detailed mechanical characteristics are rarely available in the literature. Moreover, many concrete-based systems require relatively long construction or repair periods and involve high implementation costs. These limitations motivated the development of LONGASF, which combines an asphalt supporting structure, WZA longitudinal concrete sleepers, and the dedicated ICOSTRUN-03 fastening system.

## 3. Longasf Ballastless Track Concept

**Figure 2:**
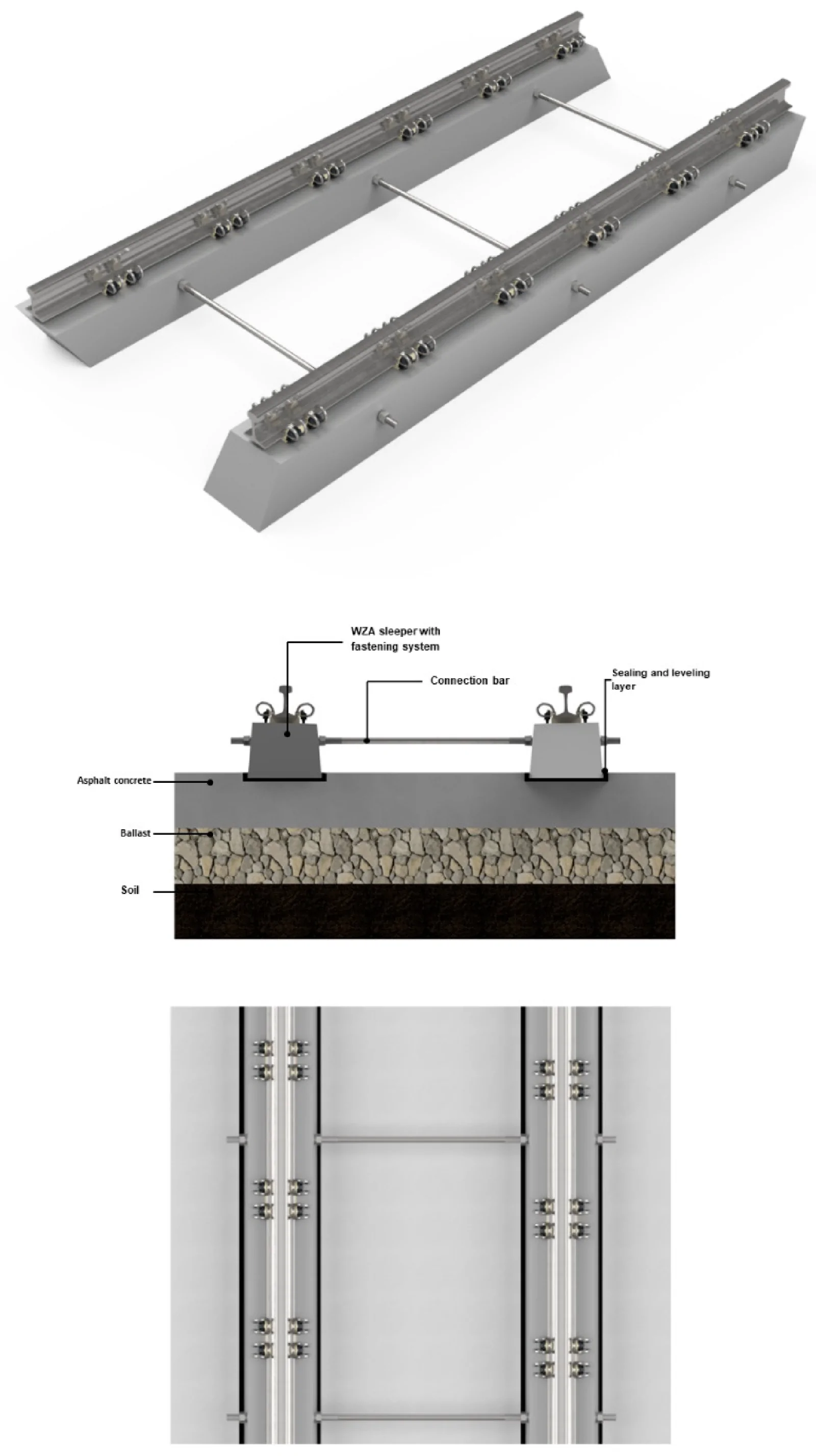
Schematic representation of the LONGASF ballastless track system.

The LONGASF ballastless track system was developed as an alternative to existing concrete- and asphalt-supported ballastless track solutions. It combines WZA longitudinal concrete sleepers, the dedicated ICOSTRUN-03 fastening system, an intermediate levelling layer, and an asphalt supporting layer. The system was intended to reduce construction and maintenance demands while retaining the principal structural and operational advantages of ballastless track. Its general configuration is presented in Figure 2.

In the LONGASF system, the WZA sleepers are installed on the asphalt supporting layer through an intermediate levelling layer. Steel transverse connectors positioned at predetermined intervals maintain the track gauge and provide lateral stability. The asphalt layer rests on a multilayer substructure designed using principles derived from pavement engineering. The structure is dimensioned to limit stresses in the asphalt layer to values comparable with those occurring in pavements subjected to heavy road traffic. A protective cover layer is placed above the structure to reduce direct solar heating and limit temperature variations in the asphalt. Recycled railway ballast may be used as the cover material, providing thermal protection and enabling the reuse of recovered track material.

### 3.1. Wza Longitudinal Sleeper

**Figure 3:**
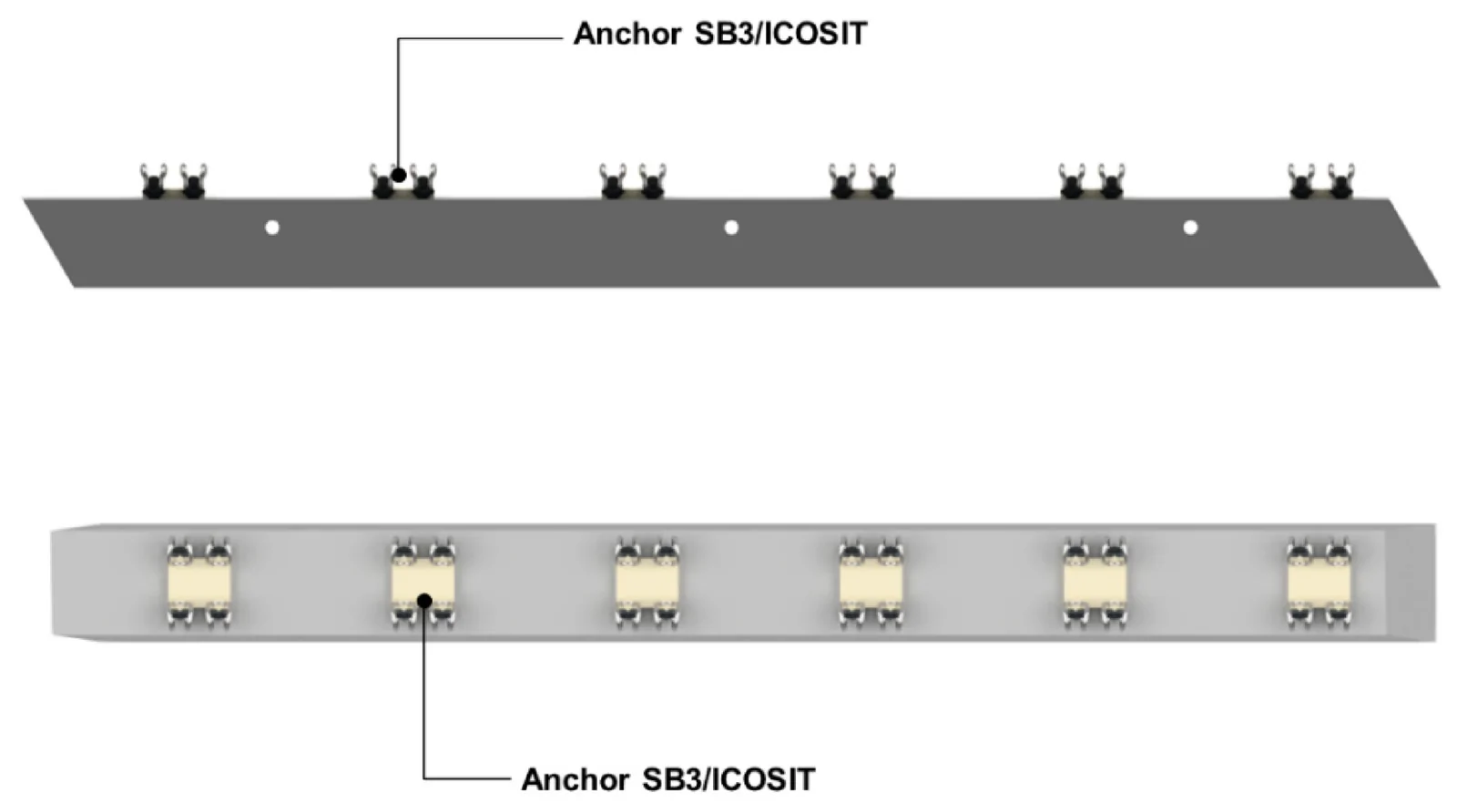
General configuration of the WZA longitudinal concrete sleeper.

The WZA sleeper (Figure 3) is a prefabricated reinforced- or prestressed-concrete beam designed for installation on either an asphalt supporting layer or conventional ballast. Longitudinal sleepers have previously been employed primarily in light-rail and tramway infrastructure, whereas their application in heavy railway systems remains limited. The WZA sleeper was specifically developed for the load-transfer conditions associated with asphalt-supported track. Adjacent sleeper sections are connected using elastic elements, while the inclined sleeper ends facilitate stress redistribution and the construction of transition zones between track structures with different stiffness characteristics.

### 3.2. Icostrun-03 Fastening System

**Figure 4:**
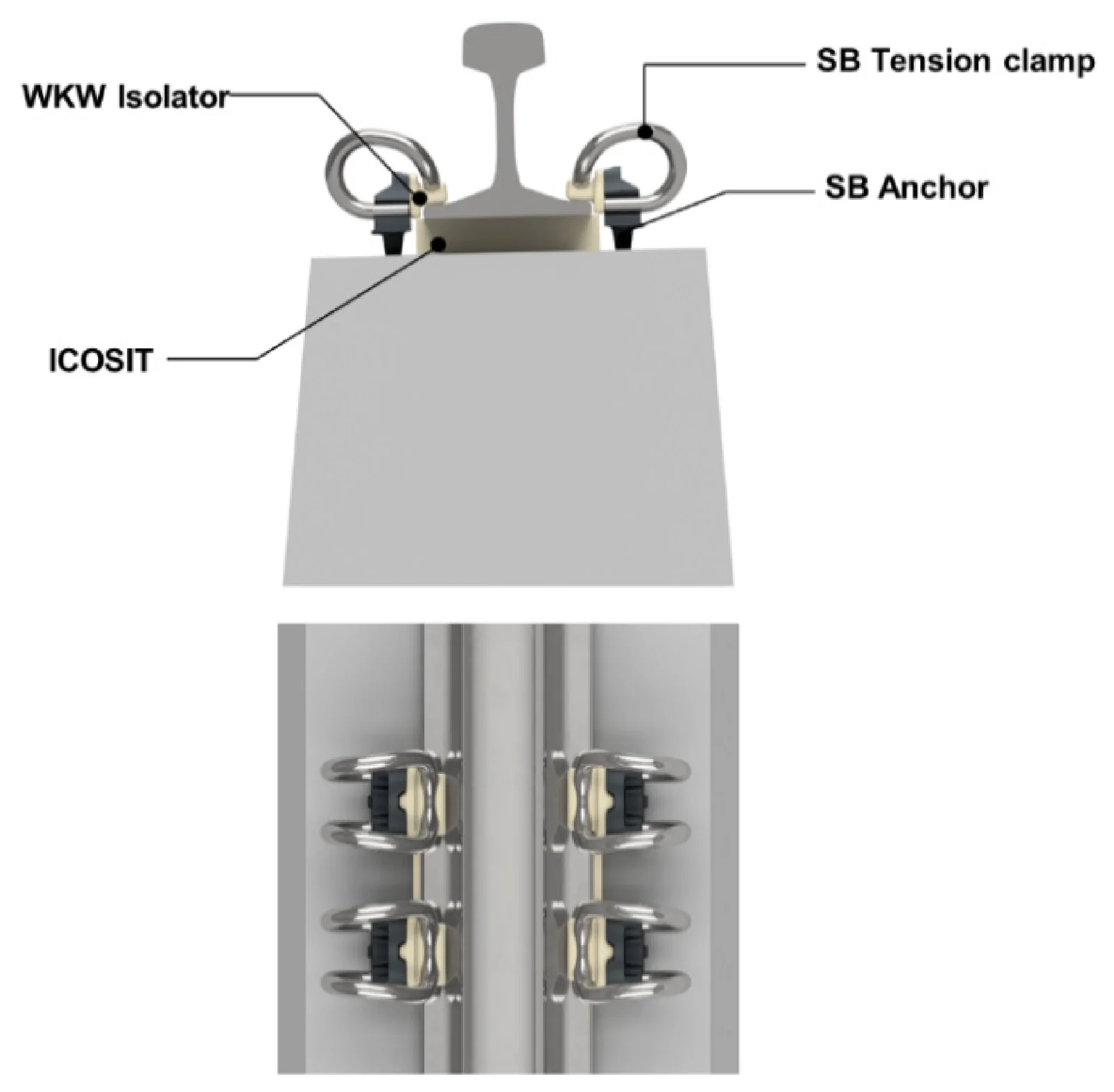
General configuration of the ICOSTRUN-03 fastening system designed for use with the WZA longitudinal sleeper.

The ICOSTRUN-03 fastening system (Figure 4) was developed specifically for the WZA sleeper configuration. It is based on the SB fastening concept and incorporates modifications derived from the earlier ICOSTRUN-02 solution, including double fastening clips and an elastic rail pad. The double-clip arrangement was intended to provide high longitudinal rail resistance under braking and traction loads, while the elastic rail pad provides the vertical flexibility required to limit dynamic forces transmitted to the sleeper and supporting structure.

### 3.3. Transition Zone Concept

**Figure 5:**
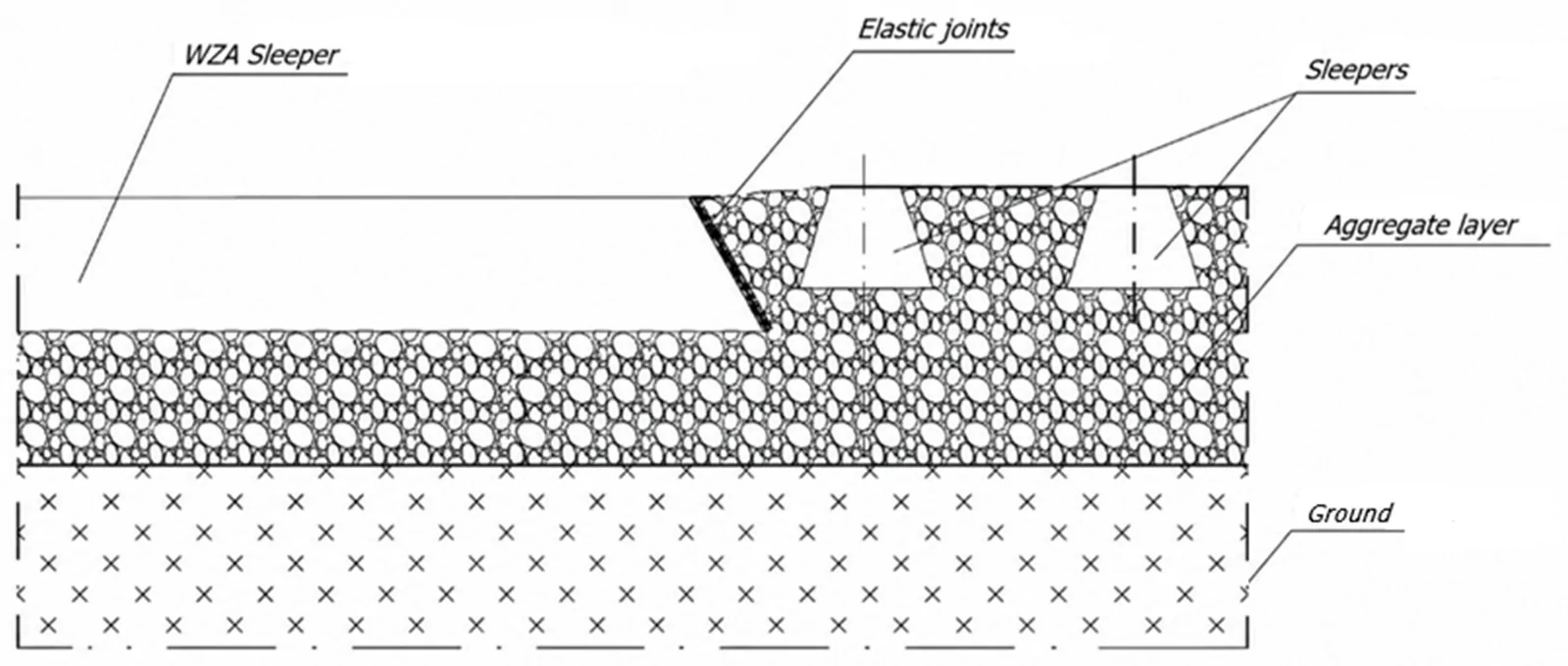
Conceptual design of the transition zone between the LONGASF ballastless track system and conventional ballasted track. The inclined end of the WZA longitudinal sleeper and the elastic mat provide a gradual stiffness transition and reduce differential settlement.

In addition to the asphalt-supported LONGASF configuration, a variant with WZA longitudinal sleepers installed on conventional ballast was considered. This variant enabled the development of a transition zone between the ballastless LONGASF system and conventional ballasted track. Such a zone is required to mitigate abrupt changes in stiffness, settlement, and deformation at the interface between the two track forms. Because ballast responds differently beneath transverse and longitudinal sleepers, an elastic mat was introduced beneath the inclined end of the WZA sleeper. The mat and the gradual change in sleeper geometry were intended to reduce differential settlement and local stress concentrations while providing a smoother transition in track stiffness. The conceptual design of the transition zone is shown in Figure 5.

The principal components of the test setup included the loading actuator, the rail section, the ICOSTRUN-03 fastening assembly, the WZA longitudinal sleeper, and the displacement-measurement system. The load was applied to the rail, while its displacement relative to the supporting structure was recorded to determine the vertical stiffness of the fastening assembly.

## 4. Experimental Characterization and Modelling of the Icostrun-03 Fastening System

### 4.1. Experimental Investigation

The mechanical properties of the ICOSTRUN-03 fastening system were determined experimentally in accordance with the requirements of EN 13481 and related standards [38,39,40,41,42]. The test programme included:

- Longitudinal rail resistance;
- Vertical stiffness determination, including dynamic rail and sleeper displacement measurements;
- Repeated loading tests;
- Transverse rail resistance.

The experiments were conducted using a Schenck Testing System, with a maximum loading capacity of 1000 kN. The applied force was measured using a type PM 1.0 K with a measuring range of 0–1000 kN and an accuracy of 0.5%. Rail displacements relative to the supporting structure were recorded using HBM inductive displacement sensors displacement transducers with a measuring range of 0–50 mm and an accuracy of 0.001 mm. The signals were recorded using a Catman Easy AP at a sampling frequency of 0–200 Hz. For the repeated-loading test, the load was applied using Schenck Testing System over the range of 20–280 kN at a frequency of 4–5 Hz. The equipment configuration was adjusted to the loading direction required for the vertical-stiffness, longitudinal-resistance, and transverse-resistance tests.

**Figure 6:**
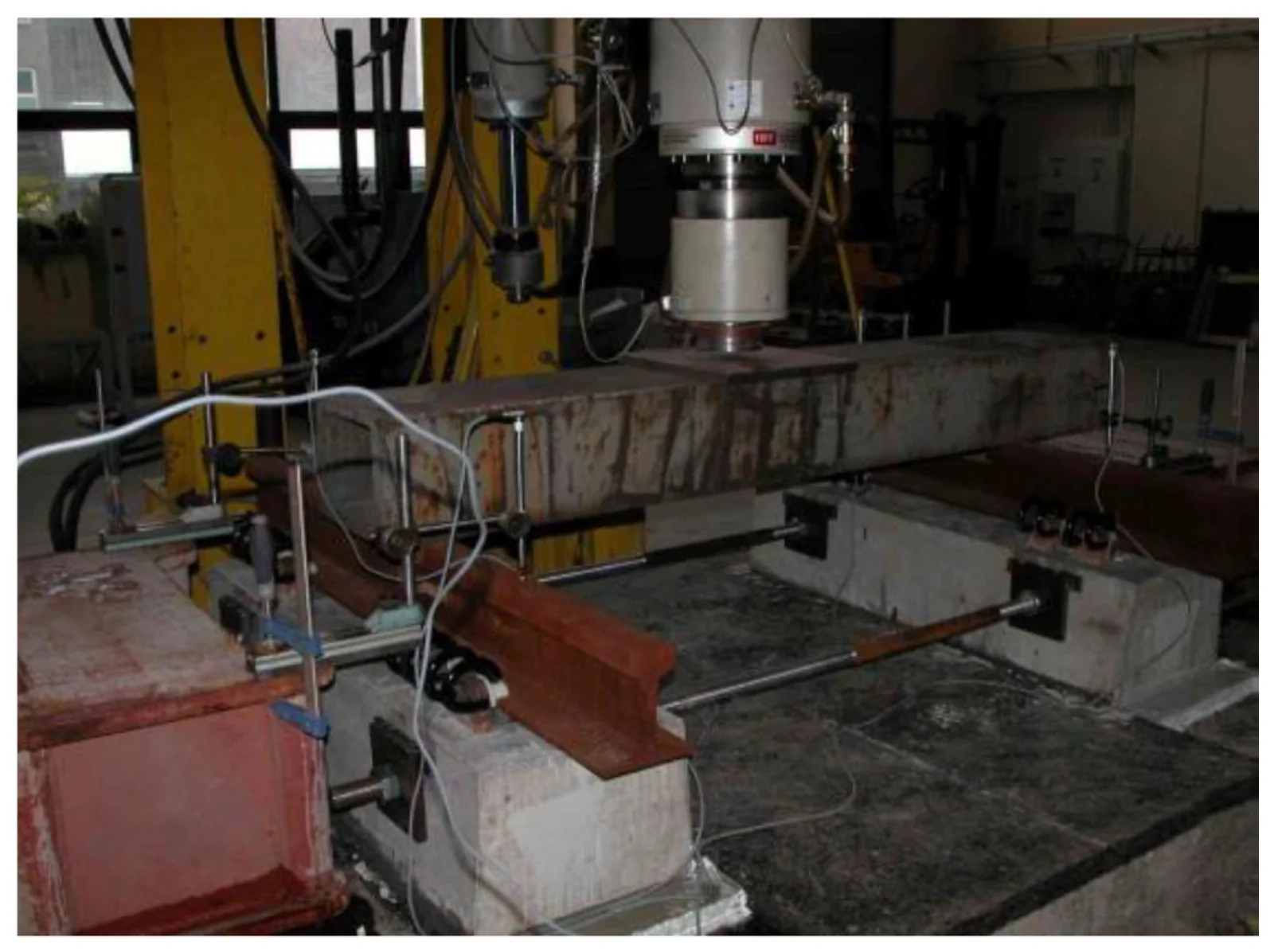
Experimental setup used for determining the vertical stiffness of the fastening system.

**Figure 7:**
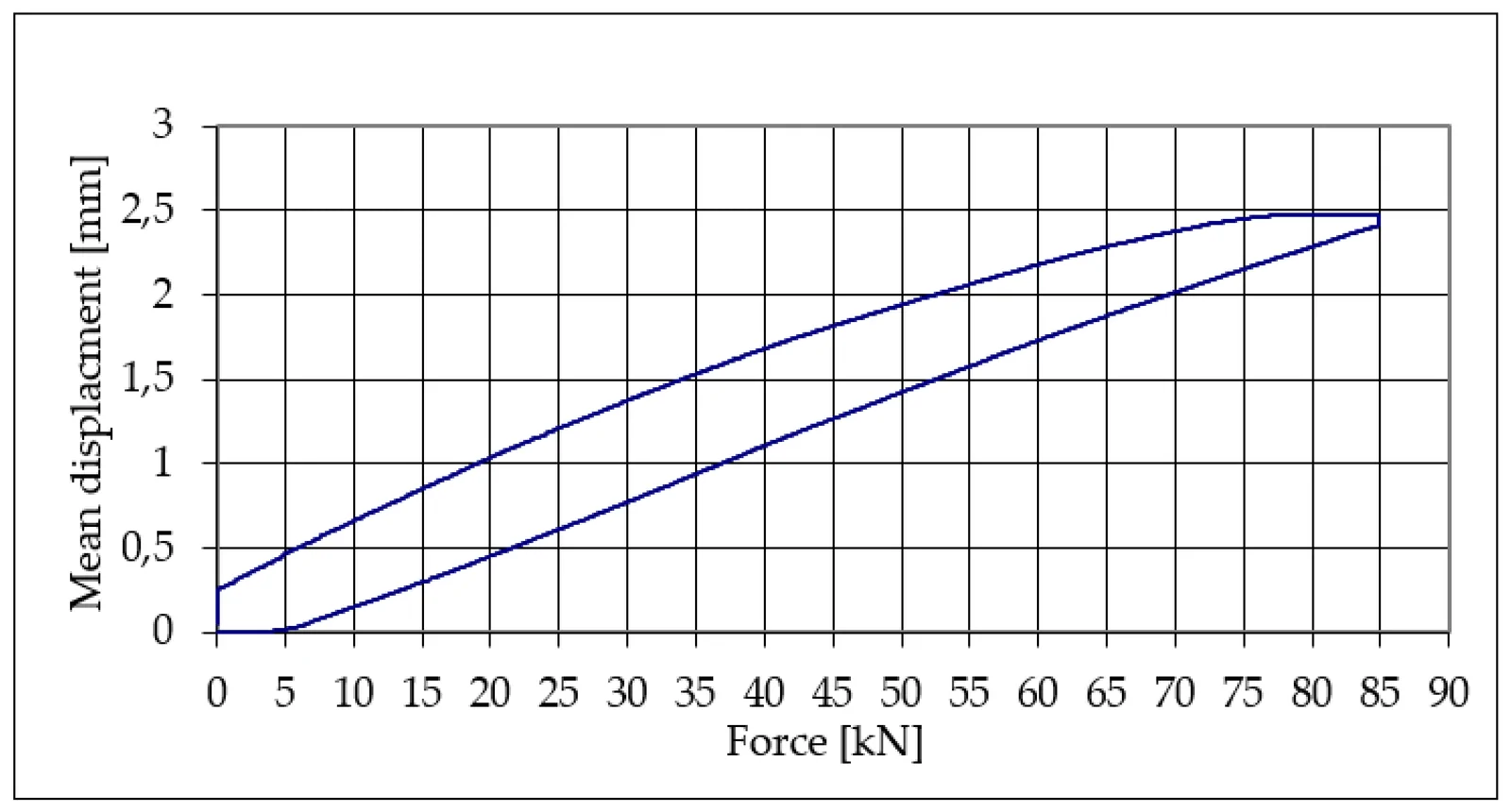
Vertical rail displacement obtained during the stiffness test.

**Figure 8:**
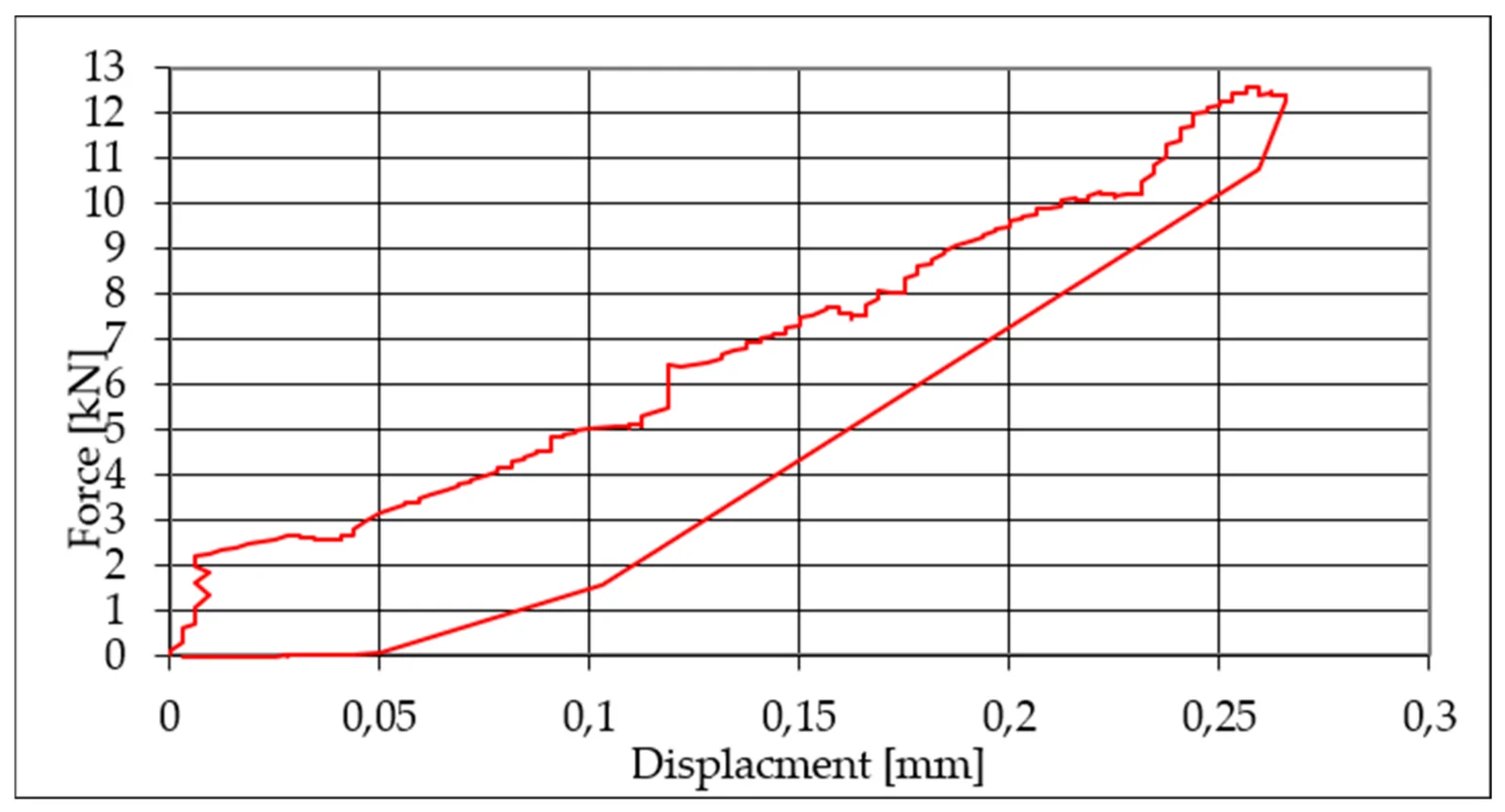
Longitudinal resistance force–displacement relationship.

Figure 6 presents an example of the test setup used for determining the vertical stiffness of the LONGASF ballastless track equipped with the ICOSTRUN-03 fastening system. Figure 7 and Figure 8 show the representative results obtained during the experimental programme, namely the vertical rail displacement recorded during the stiffness test and the longitudinal resistance force–displacement relationship, respectively. The tests were conducted indoors. In the test setup, the asphalt layer was covered with approximately 0.30 m of aggregate, placed between and outside the longitudinal sleepers.

Preliminary repeatability tests were performed on a single ICOSTRUN-03 specimen designated LB/28/12-01. Four repeated measurements were conducted for longitudinal rail resistance, three for vertical stiffness, and five for transverse rail resistance. These measurements represent technical repetitions on the same specimen rather than independent specimen replicates. Therefore, the calculated standard deviations characterize measurement repeatability under the applied laboratory conditions and should not be interpreted as estimates of between-specimen variability (Table 1).

The results of the laboratory tests are summarized in Table 2.

The repeated-loading programme comprised 1,250,000 cycles representing tangent-track operation, followed by 1,050,000 cycles representing operation in a curve. The total number of applied cycles was 2,300,000. The longitudinal rail resistance determined according to PN-EN 13146-1:2003 [39] remained above 12.6 kN throughout the programme. The value obtained using the special procedure decreased from 26.5 to 24.7 kN, corresponding to a reduction of approximately 6.8%. The vertical stiffness increased from 36.3 to 43.6 MN/m, corresponding to a change of approximately 20.1%. Both changes remained within the acceptance limits adopted in the research programme.

Preliminary tests of the ICOSTRUN-03 fastening system produced a mean longitudinal rail resistance of 12.55 kN based on four tests, a mean vertical stiffness of 36.3 MN/m based on three tests, and a mean transverse rail resistance of 36.5 kN at 1 mm displacement based on five tests.

The source documentation provides individual repeated measurements for the preliminary characterization, but only aggregate values for the measurements performed after cyclic loading. Consequently, standard deviations and confidence intervals could not be determined for the post-loading results. This limitation should be considered when interpreting the observed changes and the use of the measured values as deterministic input parameters in the numerical models.

### 4.2. Numerical Representation of the Fastening System

**Figure 9:**
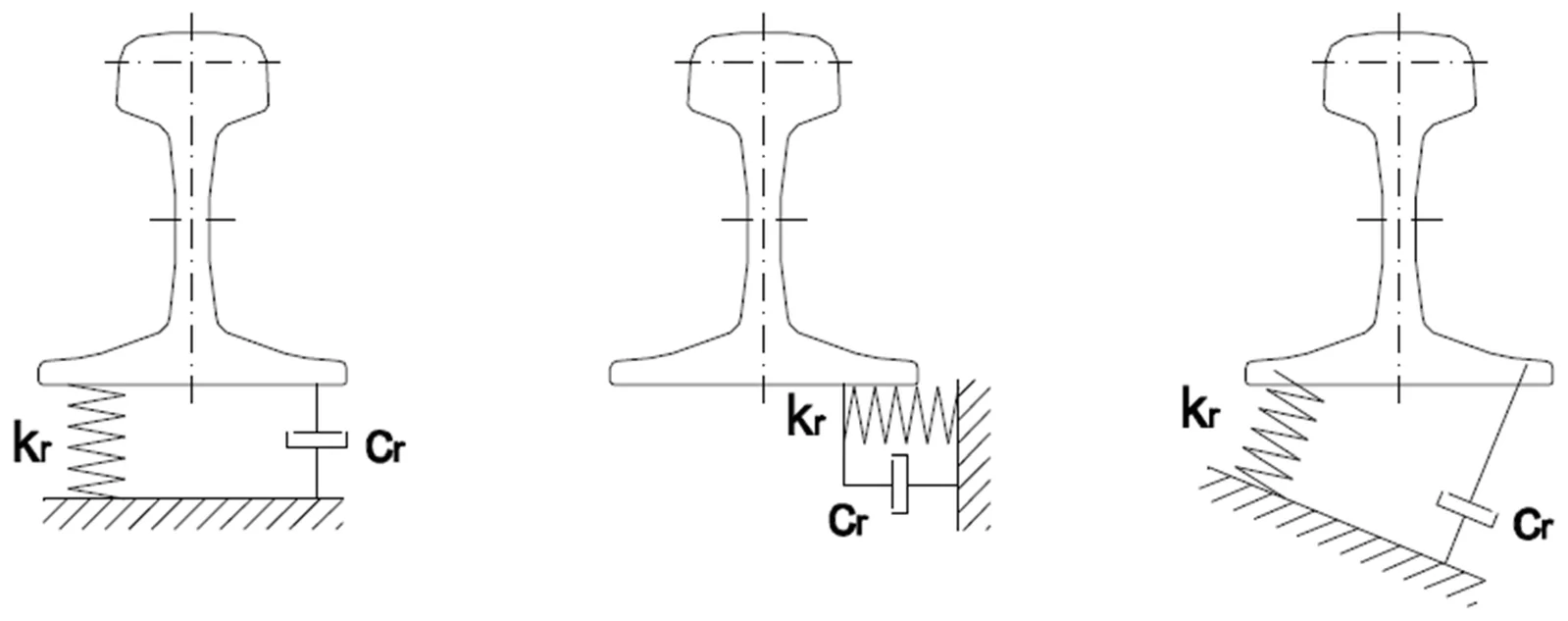
Physical representation of the fastening system using viscoelastic elements acting in the vertical, transverse, and longitudinal directions.

The experimental force–displacement characteristics were used to develop numerical representations of the ICOSTRUN-03 fastening system. Two principal modelling approaches were adopted. In the simplified dynamic models, the fastening system was represented by spring-damper elements acting independently in the vertical, transverse, and longitudinal directions (Figure 9). The corresponding stiffness and viscous-damping coefficients were determined from the laboratory results and are summarized in Section 5.

For the investigated loading range, corresponding to maximum axle loads of up to 260 kN, the vertical stiffness was determined as the secant stiffness between 5 and 80 kN in accordance with the applicable PN-EN 13481 series standards [38]. Because the standards did not specify procedures for calculating transverse and longitudinal stiffness, these parameters were obtained by linear regression of the corresponding experimental force–displacement curves. The resulting coefficients were subsequently used as input parameters in the simplified numerical models.

**Figure 10:**
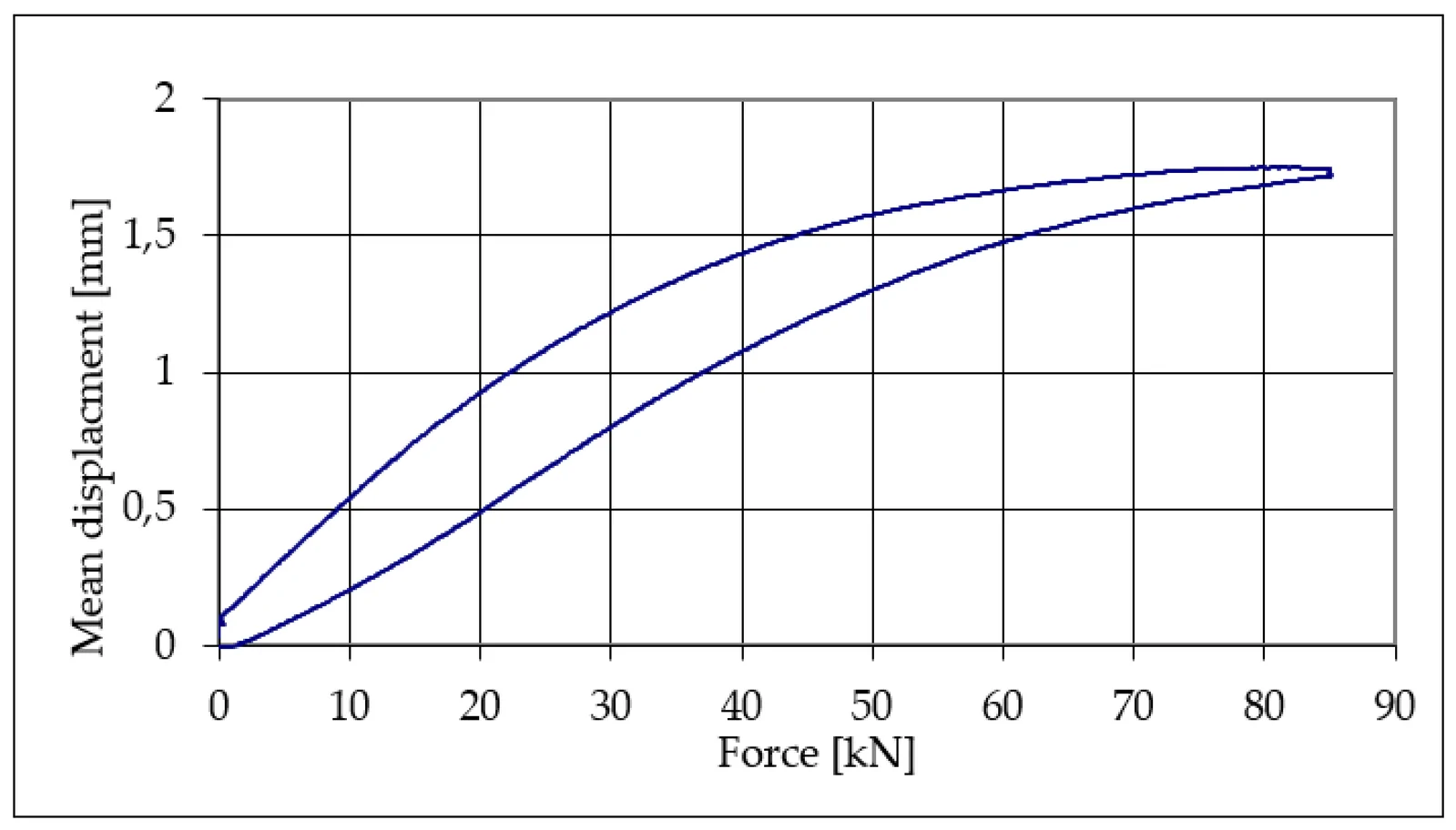
Experimental force–displacement relationship and its piecewise-linear approximation used to identify the parameters of the equivalent solid elements.

In the three-dimensional analyses, a second modelling approach was adopted in which the fastening system was represented by equivalent solid elements attached to the rail. The experimentally obtained loading and unloading curves were approximated using piecewise-linear segments (Figure 10). The mechanical properties assigned to each segment were calibrated to reproduce the measured force–displacement response.

To obtain the properties of the equivalent solid elements, the experimental force–displacement relationship was transformed into a stress–strain relationship using the assumed cross-sectional area and length of the equivalent element. For each piecewise-linear segment, the equivalent Young’s modulus *E_i_* was determined from the slope of the transformed curve. Poisson’s ratio ν_i_ was subsequently adjusted during calibration to obtain the best agreement with the experimental response. The corresponding shear modulus *G_i_* and bulk modulus *K_i_* were calculated as

*G_i_* = *E*/2(1 + *ν_i_*) (1)

*K_i_* = *E*/3(1 − 2*ν_i_*) (2)

A conceptual extension of the fastening model was also considered, in which the limiting longitudinal or transverse resistance *R* depended linearly on the current vertical rail load *Q* [43]:

*R* = *R*_0_ + *aQ* (3)

where *R*_0_ is the fastening resistance in the absence of vertical loading and *a* is an experimentally determined coefficient. This formulation was considered as a potential extension of the basic model; however, it was not used in the analyses reported in the following sections because system-specific values of *R*_0_ and *a* were not available.

## 5. Theoretical and Numerical Models of the Longasf Ballastless Track System

The mechanical response of the LONGASF ballastless track system was investigated using two complementary modelling frameworks. The first was a three-dimensional numerical model in which the rail, longitudinal sleeper, equivalent fastening elements, and supporting components were represented using elastic solid elements characterized by Young’s modulus (*E*) and Poisson’s ratio (*ν*). The second was a simplified model used primarily for dynamic analyses [43]. In the simplified model:

- The rail was represented as a beam with actual mechanical characteristics, including bending stiffness *EI_z_* (vertical direction), *EI_y_* (lateral direction), and longitudinal stiffness EA, where E is the Young’s modulus of rail steel, *I_z_* and *I_y_* are the moments of inertia about the vertical and lateral axes, respectively, and A is the rail cross-sectional area.
- The fastening system was represented by viscoelastic elements or nonlinear load–displacement characteristics.
- The longitudinal sleeper was represented using the same beam formulation as the rail, but with its own material, geometric, and mass properties. This modelling approach was adopted because the WZA sleeper is a continuous longitudinal structural member whose global response is governed primarily by axial deformation and bending in the vertical and transverse planes. The sleeper was characterized by the Young’s modulus of concrete $E_{b}=35,000\;MPa$, a cross-sectional area of $A_{b}=0.1125\;m^{2}$, and second moments of area of $I_{z}=84,375\;cm^{4}$ and $I_{y}=126,633\;cm^{4}$. Its interaction with the supporting structure was represented by distributed viscoelastic elements. Thus, the analogy with the rail concerned the mathematical beam formulation only and did not imply identical material or sectional properties.
- The support beneath the longitudinal sleeper was represented by viscoelastic elements determined on the basis of experimental investigations, including both laboratory and field measurements.

The parameters adopted for the simplified model of the LONGASF track with ICOSTRUN-03 fastening systems spaced at 0.75 m are summarized in Table 3. The fastening stiffnesses were derived from the experimental force–displacement relationships, while the viscous-damping coefficients were determined from the corresponding hysteresis loops.

For the three-dimensional model, standard linear-elastic material properties were adopted for steel, concrete, the levelling layer, and the asphalt supporting layer. The Young’s modulus of the asphalt layer was assumed to be *Ea* = 10,000 MPa. This value was adopted as a lower-bound modulus corresponding to asphalt temperatures not exceeding approximately 25–30 °C. The asphalt layer was therefore covered with an approximately 0.30 m thick protective ballast layer intended to limit direct solar heating. The effectiveness of such protection is supported by field measurements reported for an asphalt-supported track structure, in which the maximum asphalt-surface temperature reached, even when temperatures at the ballast-cover surface exceeded [44]. The value of, however, should be regarded as a modelling assumption because its temperature dependence was not investigated experimentally within the present study.

Previous investigations of railway asphalt sub-ballast mixtures have demonstrated that the stiffness modulus depends strongly on both temperature and loading frequency. Reference [45] determined the mechanical properties of railway asphalt mixtures at 10, 17, and 25 °C over a frequency range of 0.1–20 Hz and incorporated the resulting temperature-dependent properties into a dynamic track model. However, because the LONGASF analyses were performed using a constant asphalt modulus of $E_{a}=10,000\;MPa$, the present results represent a single assumed material condition rather than the full range of seasonal asphalt behaviour. A dedicated temperature-dependent sensitivity analysis is therefore required in further work.

## 6. Simplified Dynamic Analysis Under Moving Non-Inertial Loading in the Vertical Plane

The vertical response of the LONGASF track system was investigated using a simplified beam-on-elastic-foundation model. In this approach, the moving wheel loads were applied without explicitly modelling the vehicle masses and their inertial interaction with the track. Owing to the symmetry of the loading with respect to the track axis, the response of a single rail and its supporting longitudinal sleeper was considered.

In the first stage, the rail was represented as an Euler–Bernoulli beam on an equivalent elastic foundation and subjected to a moving wheel load. The rail-deflection line was determined for successive load positions and expressed in a coordinate system moving with the load. In the second stage, the calculated rail displacement was introduced as the kinematic excitation of the longitudinal sleeper. The sleeper was represented as a longitudinal beam with its own distributed mass and bending stiffness. It was connected to the rail through the vertical fastening stiffness $k_{r,z}$ and damping coefficient $c_{r,z}$, and to the supporting structure through the distributed vertical stiffness $U_{b,z}$ and damping coefficient $c_{b,z}$. The fastening parameters were determined from the laboratory force–displacement characteristics described in Section 4.2.

The calculations were performed for train speeds ranging from 0 to 250 km/h and for four values of the foundation modulus: $C_{b}=75$, 100, 150, and 200 MN/m^3^.

For the effective support width $b_{eff}=0.90\;m$ adopted in the LONGASF model, the corresponding distributed vertical foundation stiffness was calculated as

$$U_{b,z}=C_{b}b_{eff},$$

giving

$$U_{b,z}=67.5,\;90,\;135,\;\mathrm{and}\;180\;MN/m^{2}.$$

**Figure 11:**
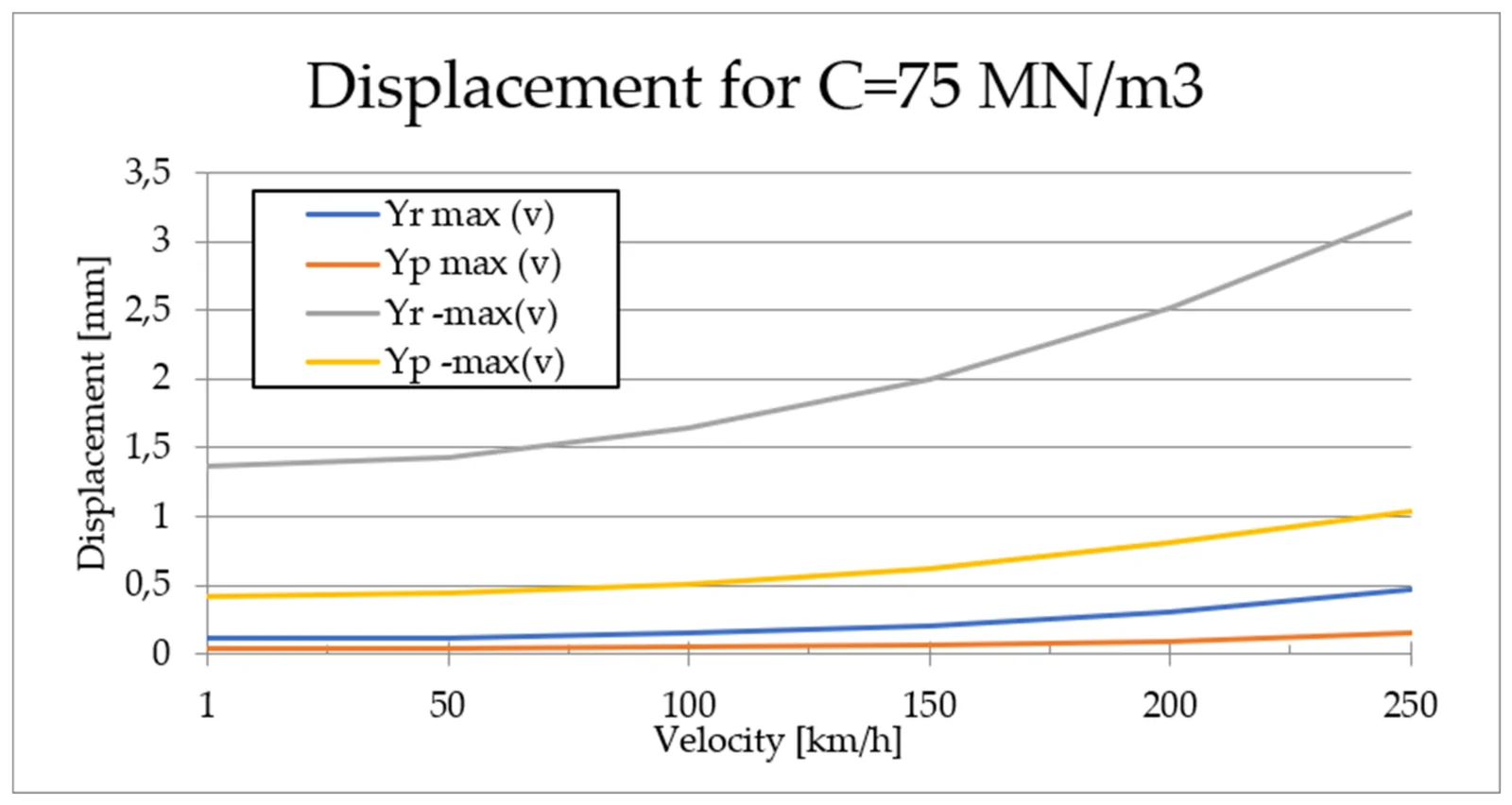
Maximum rail and sleeper displacements in the LONGASF track system for Cb = 75 MN/m^3^.

**Figure 12:**
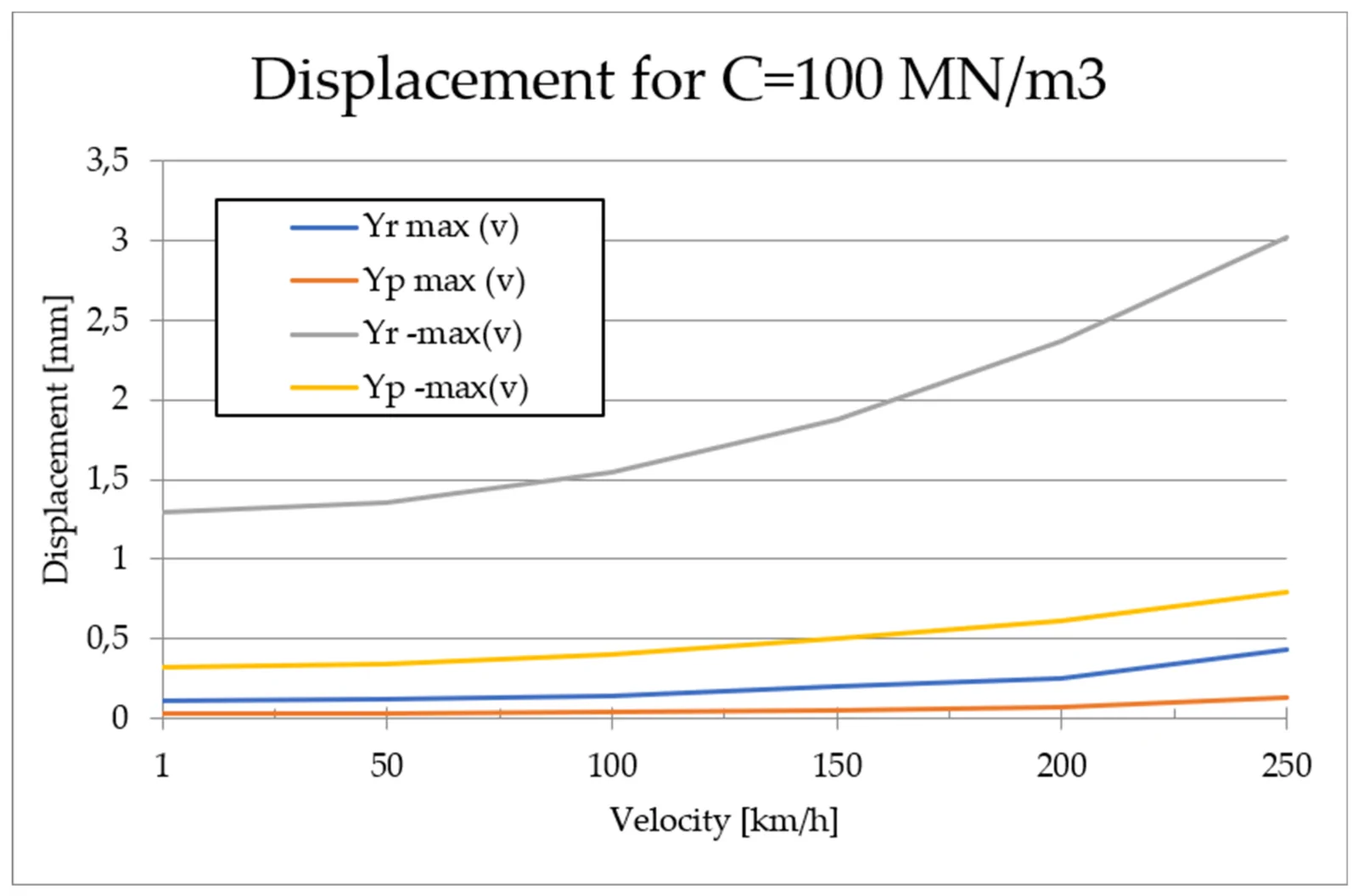
Maximum rail and sleeper displacements in the LONGASF track system for *Cb* = 100 MN/m^3^.

**Figure 13:**
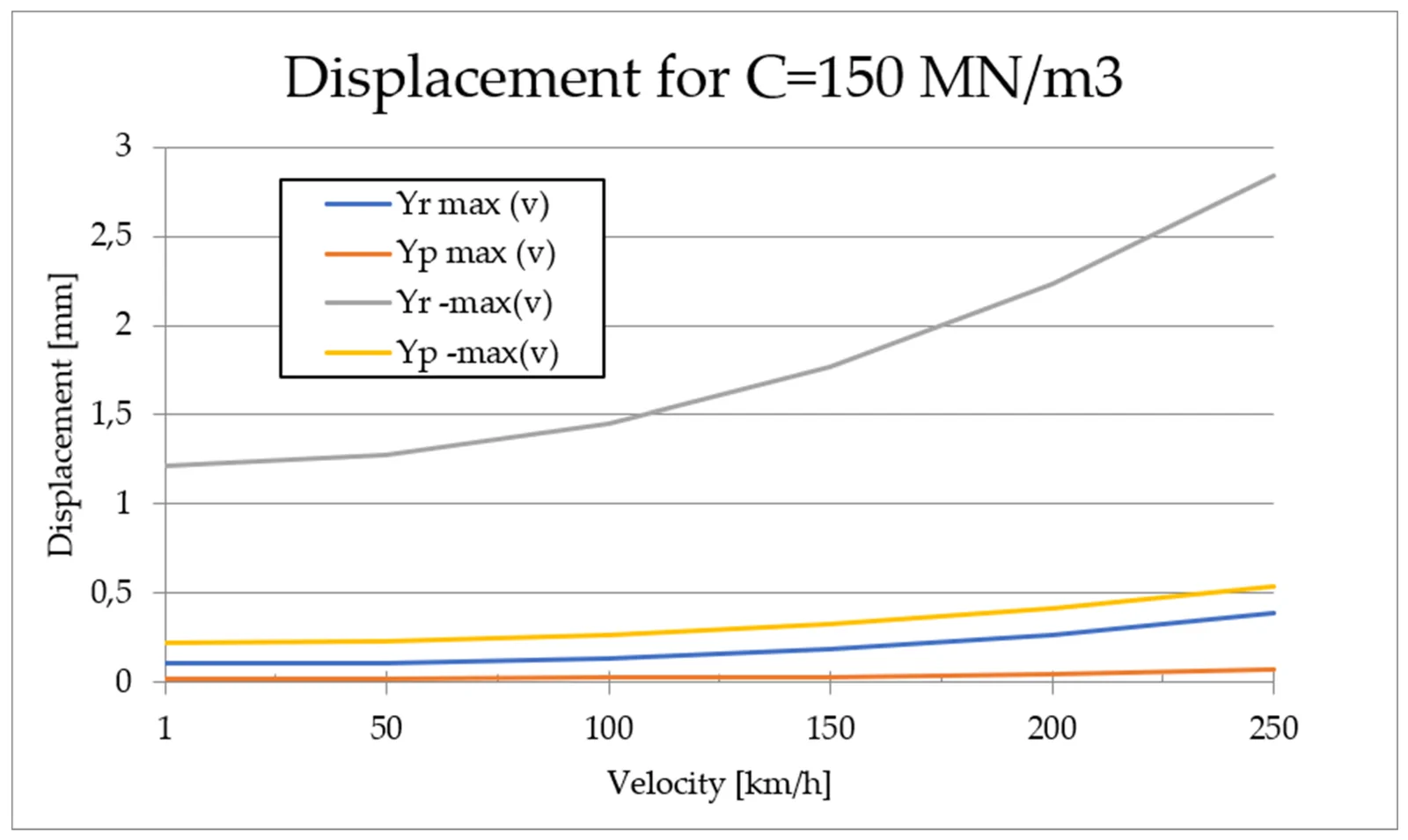
Maximum rail and sleeper displacements in the LONGASF track system for *Cb* = 150 MN/m^3^.

**Figure 14:**
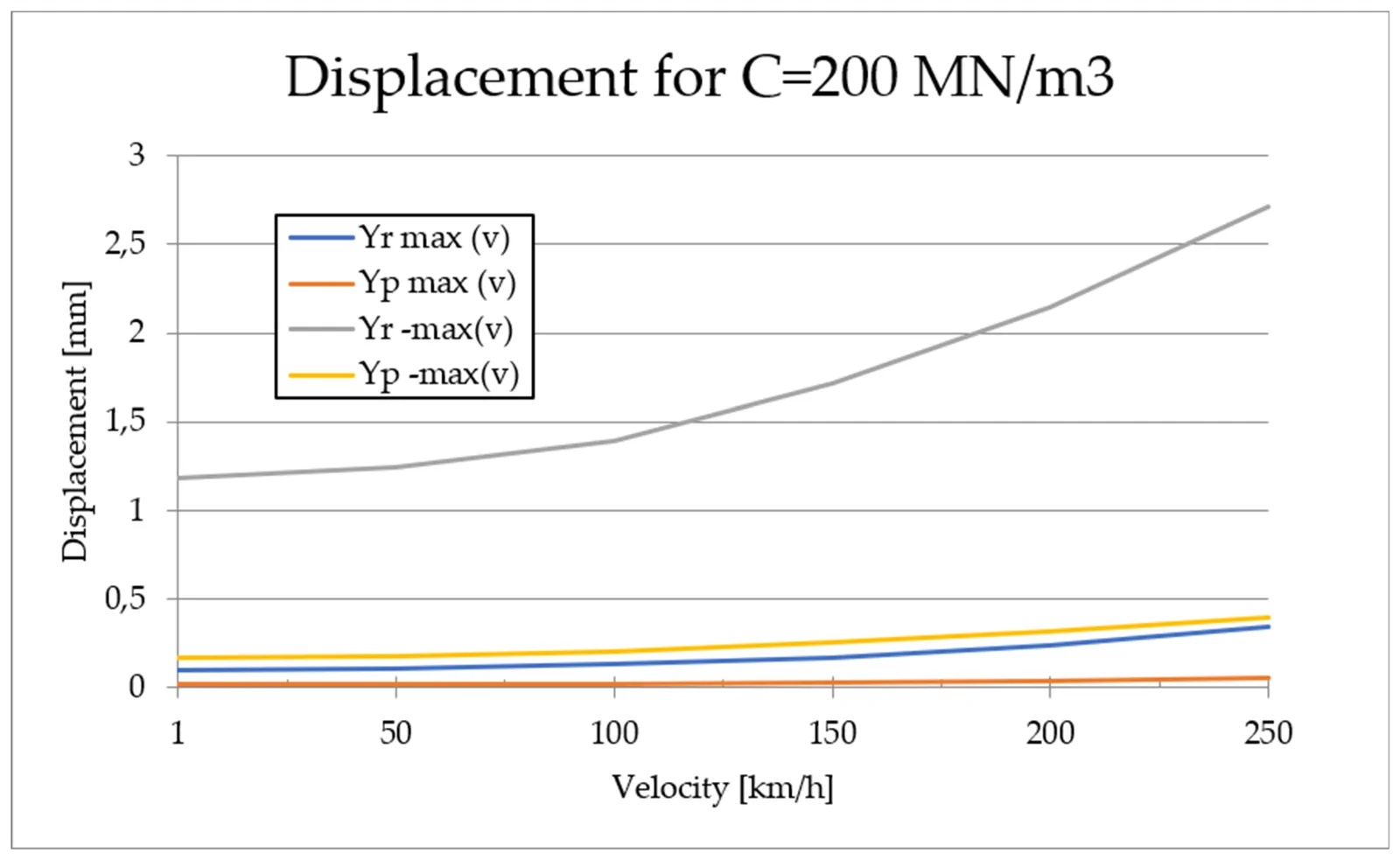
Maximum rail and sleeper displacements in the LONGASF track system for *Cb* = 200 MN/m^3^.

**Figure 15:**
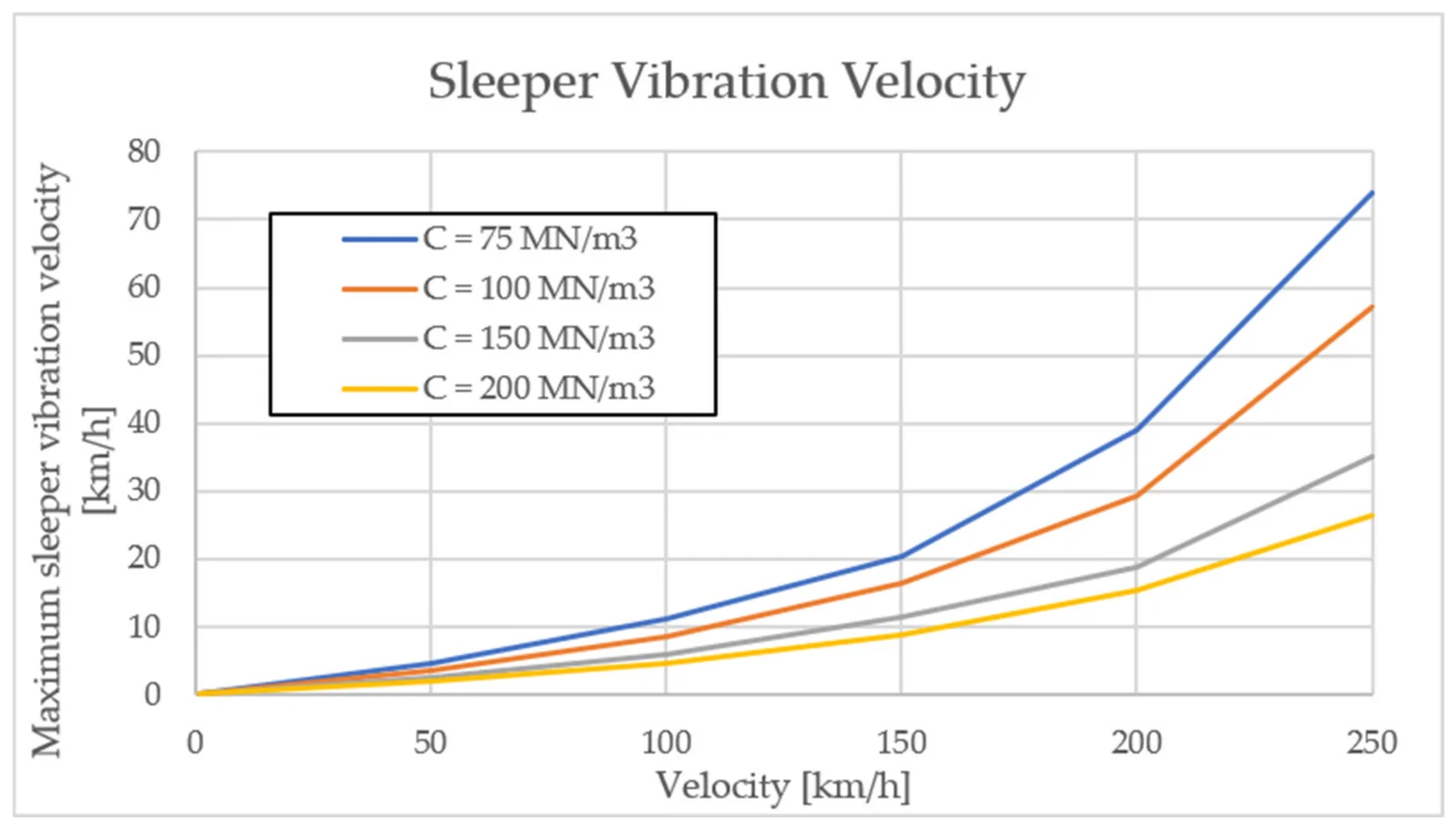
Maximum vibration velocity of longitudinal sleepers in the LONGASF track system as a function of train speed for different values of the foundation modulus.

**Figure 16:**
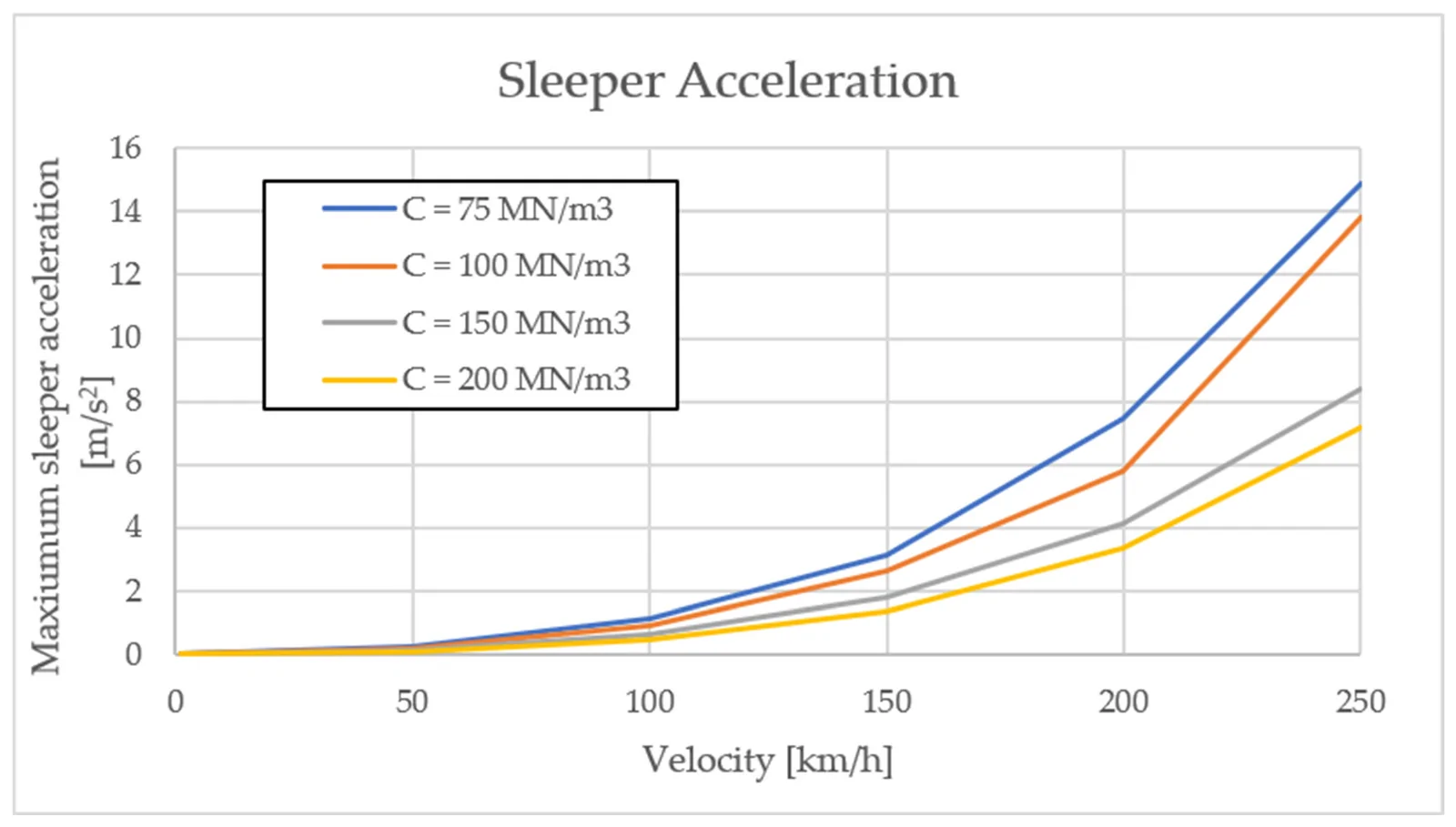
Maximum acceleration of longitudinal sleepers in the LONGASF track system as a function of train speed for different values of the foundation modulus.

For each combination of train speed and foundation modulus, the displacement responses of the rail and longitudinal sleeper were calculated. Sleeper vibration velocity and acceleration were obtained as the first and second time derivatives of the calculated displacement response, respectively. The maximum upward and downward displacements are presented in Figure 11, Figure 12, Figure 13 and Figure 14, while the maximum sleeper vibration velocities and accelerations are presented in Figure 15 and Figure 16.

Because the adopted formulation represents the train load as a prescribed moving force, the calculated sleeper accelerations should be interpreted only as response indicators within a comparative parametric analysis. They cannot be used directly to assess vehicle running safety, wheel–rail contact stability, passenger comfort, or the permissible operating speed of the LONGASF system.

The influence of train speed on the maximum vibration velocity of the longitudinal sleeper is presented in Figure 15 for the entire range of foundation stiffness values considered in the study.

Similarly, the influence of train speed on the maximum acceleration of the longitudinal sleepers is presented in Figure 16.

Considering the vertical response of the LONGASF track system and assuming an average foundation stiffness of *Cb* = 100 MN/m^3^, the screening criteria adopted in this study were based on recommendations reported in the literature for high-performance railway track structures [46,47,48]:

(a) The Maximum Vertical Displacement of the Sleeper Should Not Exceed 1 mm;
(b) The Vibration Velocity of the Sleeper Should Remain Below 50 mm/s;
(c) The vibration acceleration of the sleeper should remain below 1 g (9.81 m/s^2^).

For the representative foundation modulus of *Cb* = 100 MN/m^3^, the adopted displacement criterion was satisfied throughout the investigated speed range. However, both the vibration-velocity limit of 50 mm/s and the acceleration screening limit of 9.81 m/s^2^ were exceeded at the highest analysed speed. Because the simplified model does not include vehicle inertia, suspension dynamics, or coupled wheel–rail interaction, these exceedances identify a range requiring further investigation and cannot be used to determine the permissible operating speed of the LONGASF system.

## 7. Quasi-Static Finite Element Analysis of the Longasf Track Structure

The numerical models described in the preceding sections were combined with the experimentally determined mechanical characteristics of the fastening system. In the linear-elastic analyses, experimentally obtained secant stiffness values were used. In the nonlinear analyses, the measured force–displacement curves were approximated separately along the loading and unloading paths. The rail was represented as a three-dimensional elastic body with its actual geometry and material properties. The fastening system was represented by equivalent three-dimensional elements calibrated to reproduce its measured mechanical response, whereas the sleeper and supporting structure were represented by equivalent elastic constraints.

The analyses were performed under quasi-static loading conditions. In this study, the term *quasi-static* refers to static loads increased by coefficients representing selected dynamic effects of train operation, without explicitly modelling the inertia of the vehicle. For vertical loading, the static wheel load of the EP09 locomotive was multiplied by the dynamic amplification factor corresponding to a train speed of approximately 230 km/h:

Q_d_ = 2Q_st_ (4)

For transverse loading, an uncompensated lateral acceleration of 1.2 m/s^2^, was assumed. The transverse force acting on one rail was therefore calculated as

Y = 0.244Q_st_ (5)

For longitudinal loading, a braking intensity of 0.2 was adopted. The corresponding longitudinal force acting on one rail was

T = 0.4Qst = 0.2Qd(6)

For the LONGASF model, the maximum load levels applied during the laboratory investigations were used.

**Figure 17:**
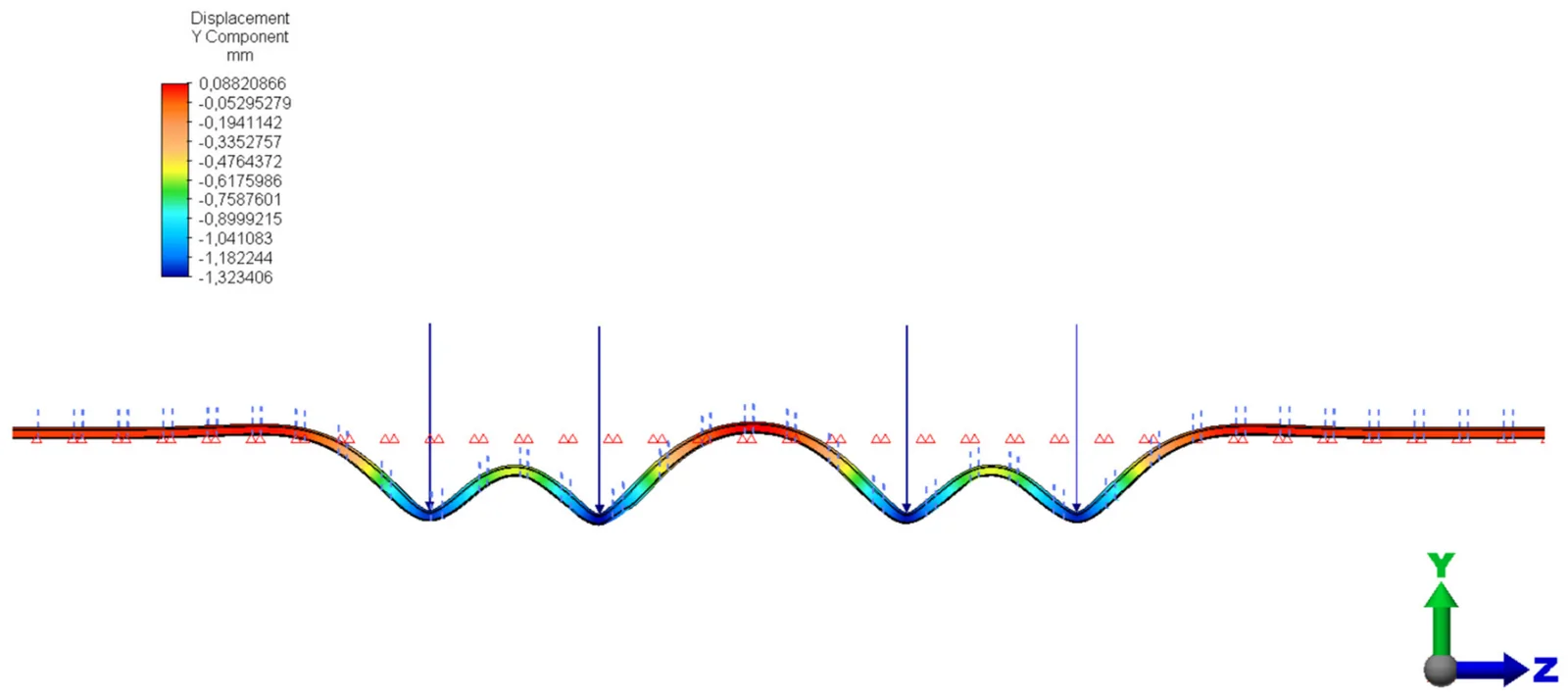
Vertical displacements for *k*_p,z_ = 131.25 kN/mm.

**Figure 18:**
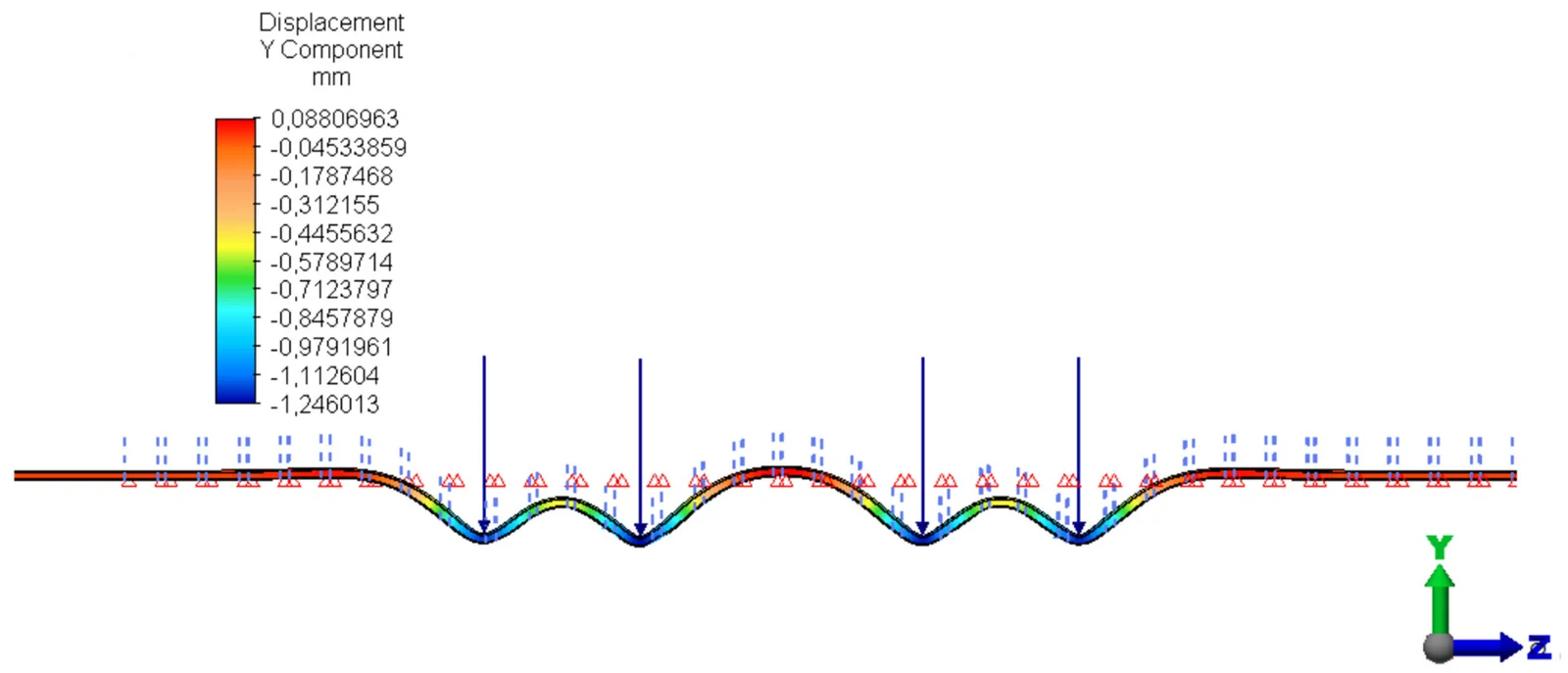
Vertical displacements for *k*_p,z_ = 196.9 kN/mm.

In the vertical quasi-static analyses, the fastening system and the support beneath the longitudinal sleeper were represented by separate stiffness parameters. The experimentally determined vertical fastening stiffness used in the calculations was $k_{r,z}=43.6\;kN/mm$. Two values of the equivalent vertical support stiffness beneath the longitudinal sleeper were considered: $k_{p,z}=131.25\;kN/mm$ and $k_{p,z}=196.9\;kN/mm$. Thus, the latter values represent two variants of the supporting structure and should not be interpreted as fastening stiffnesses. The calculated vertical displacements are presented in Figure 17 and Figure 18.

**Figure 19:**
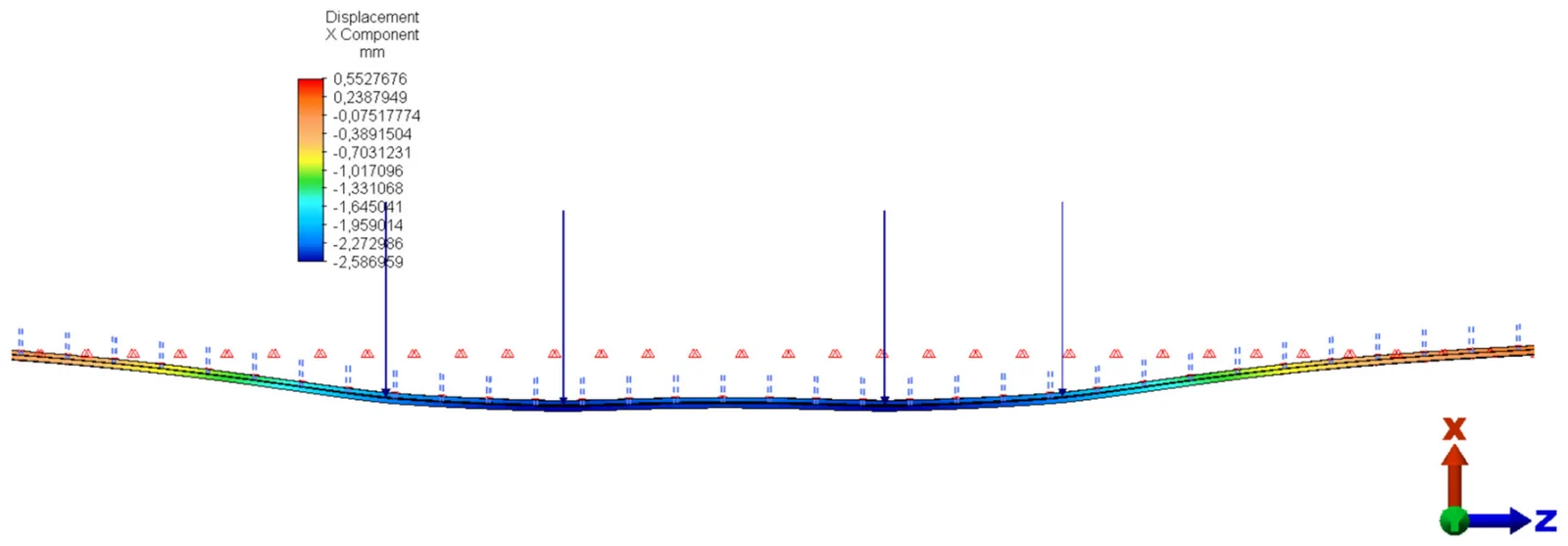
Transverse displacements for *k*_p,y_ = 5 kN/mm.

**Figure 20:**
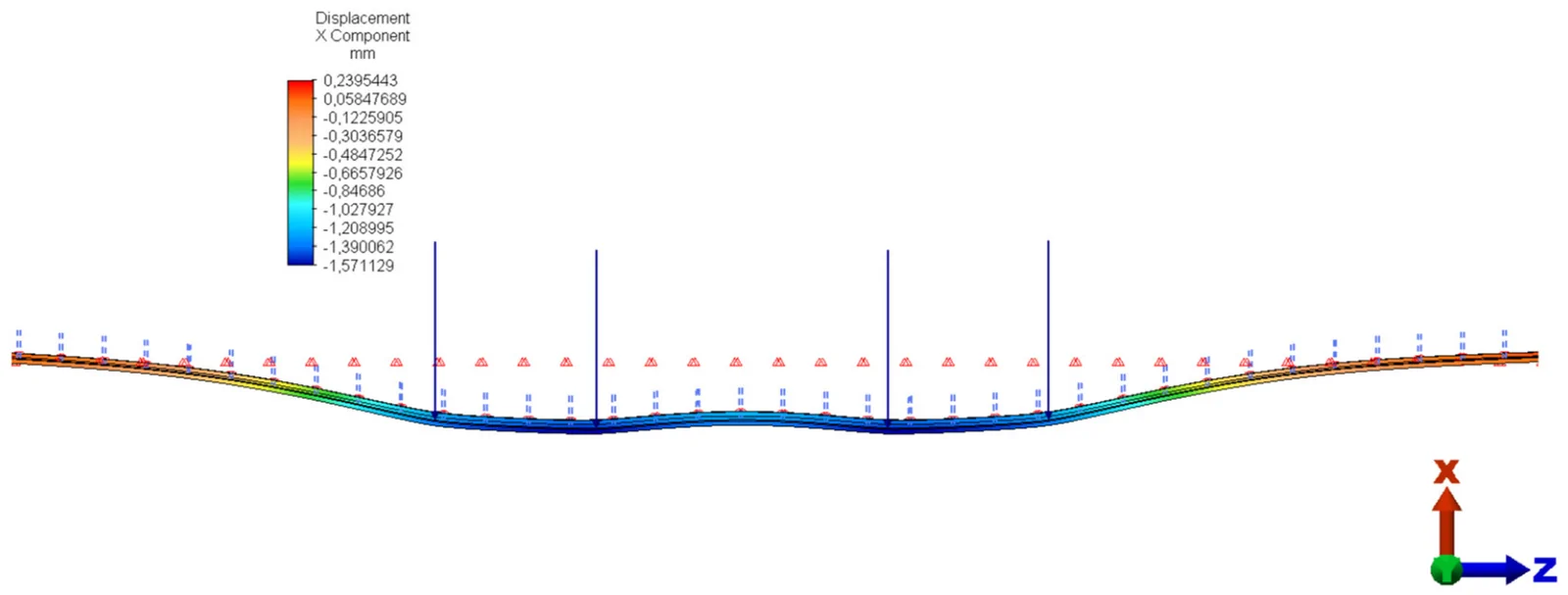
Transverse displacements for *k*_p,y_ = 10 kN/mm.

The maximum vertical displacement reached 1.323 mm for *k*_p,z_ = 131.25 kN/mm and 1.246 mm for *k*_p,z_ = 196.9 kN/mm. The corresponding transverse displacements are presented in Figure 19 and Figure 20.

The maximum transverse displacement reached 2.587 mm for *k_p,y_* = 5 kN/mm and 1.571 mm for *k_p,y_* = 10 kN/mm. To investigate the influence of neighbouring fastening systems, a detailed three-dimensional finite element model was developed in Autodesk Simulation Multiphysics based on the laboratory specimen. The model consisted of equivalent three-dimensional brick elements and actuator elements reproducing the clamping force applied by the fastening clips. The soil beneath the asphalt layer was represented using spring elements. A conservative foundation modulus of C_b_ = 75 MN/m^3^ was adopted, while the ballast cover layer was neglected.

**Figure 21:**
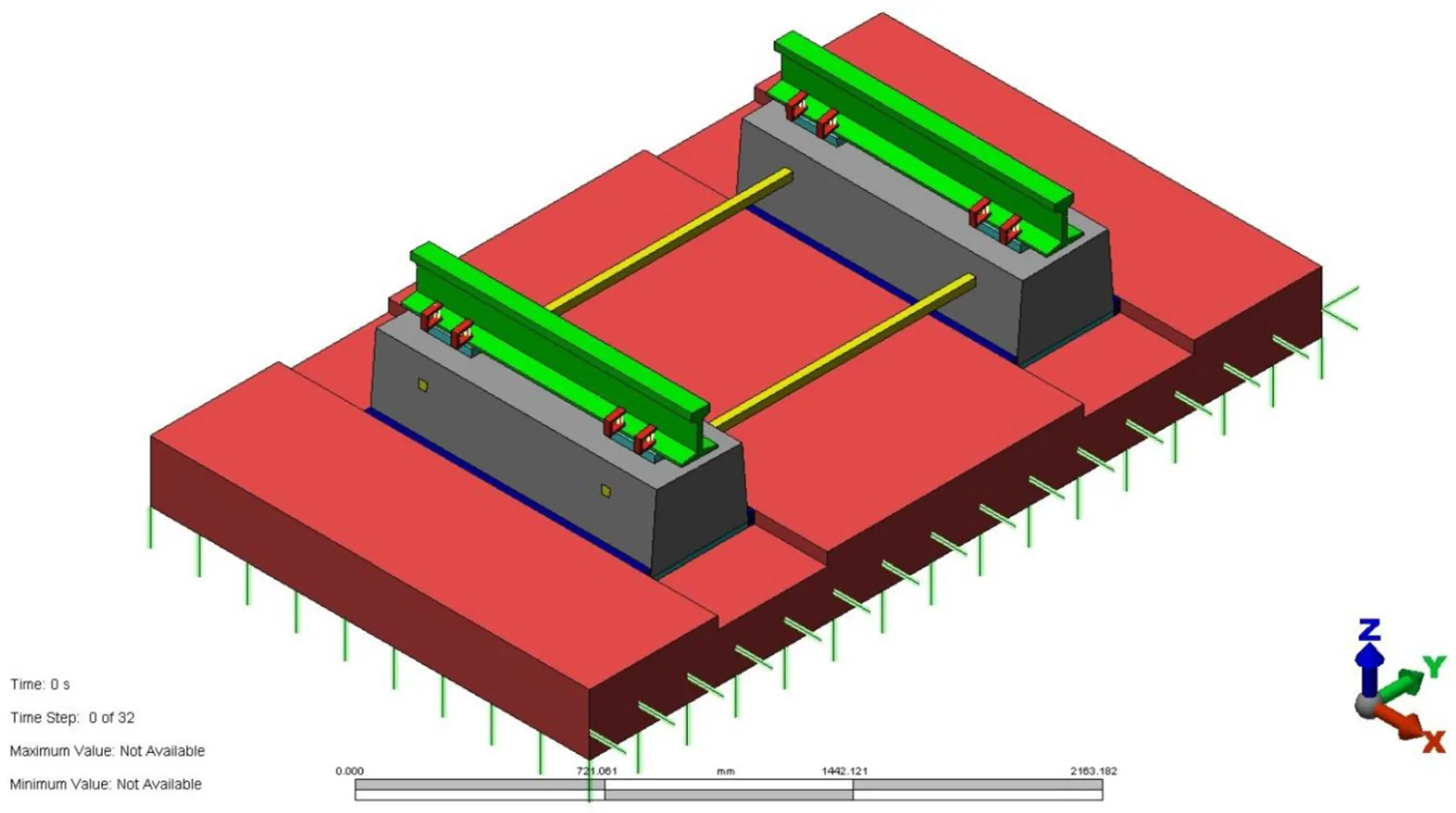
Three-dimensional numerical model of the laboratory specimen, including the rail, equivalent fastening elements, WZA longitudinal sleeper, asphalt supporting layer, and spring representation of the subgrade.

The schematic representation of the rail–fastening–sleeper system adopted in the analyses is presented in Figure 9. In the detailed three-dimensional model shown in Figure 21, the rail, WZA longitudinal sleeper, levelling layer, and asphalt supporting layer were represented by three-dimensional solid elements. The fastening system was represented by equivalent elastic elements calibrated using the experimentally determined force–displacement characteristics. Actuator elements reproduced the clamping action of the fastening clips, while the soil beneath the asphalt layer was represented by discrete spring elements. A foundation modulus of $Cb=75\;MN/m^{3}$ was adopted as the conservative support condition. As in the laboratory configuration, the protective ballast cover was not included in this model.

**Figure 22:**
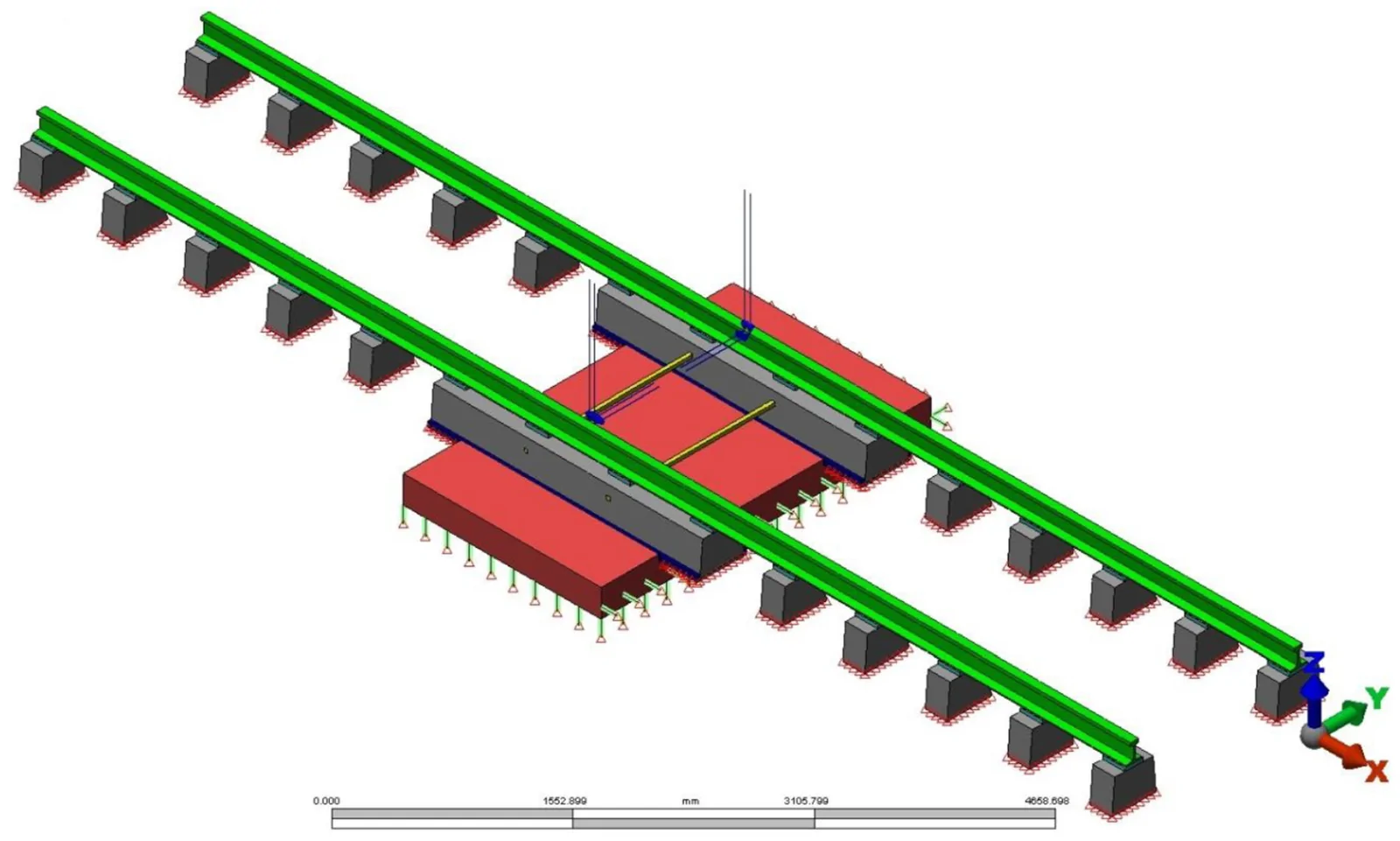
Longitudinally extended three-dimensional model including adjacent fastening regions.

The initial model reproduced the extent of the laboratory specimen and was used to examine the local response of a single fastening region. It was subsequently extended in the longitudinal direction by adding adjacent rail-support and fastening regions, as shown in Figure 22. The extended model was used to determine how load redistribution through neighbouring fastening systems affected the calculated displacement field.

**Figure 23:**
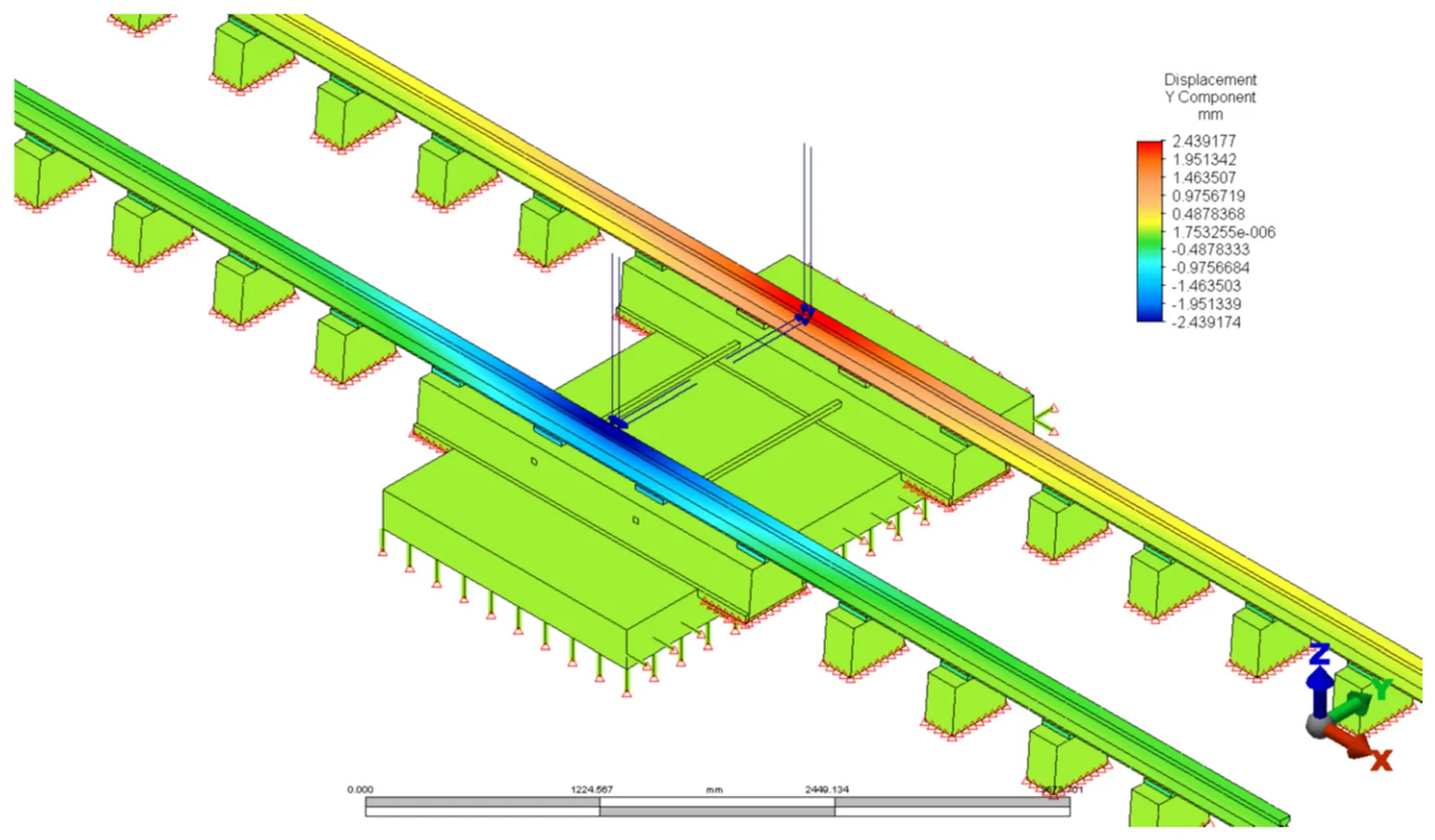
Horizontal displacements of the LONGASF track model.

**Figure 24:**
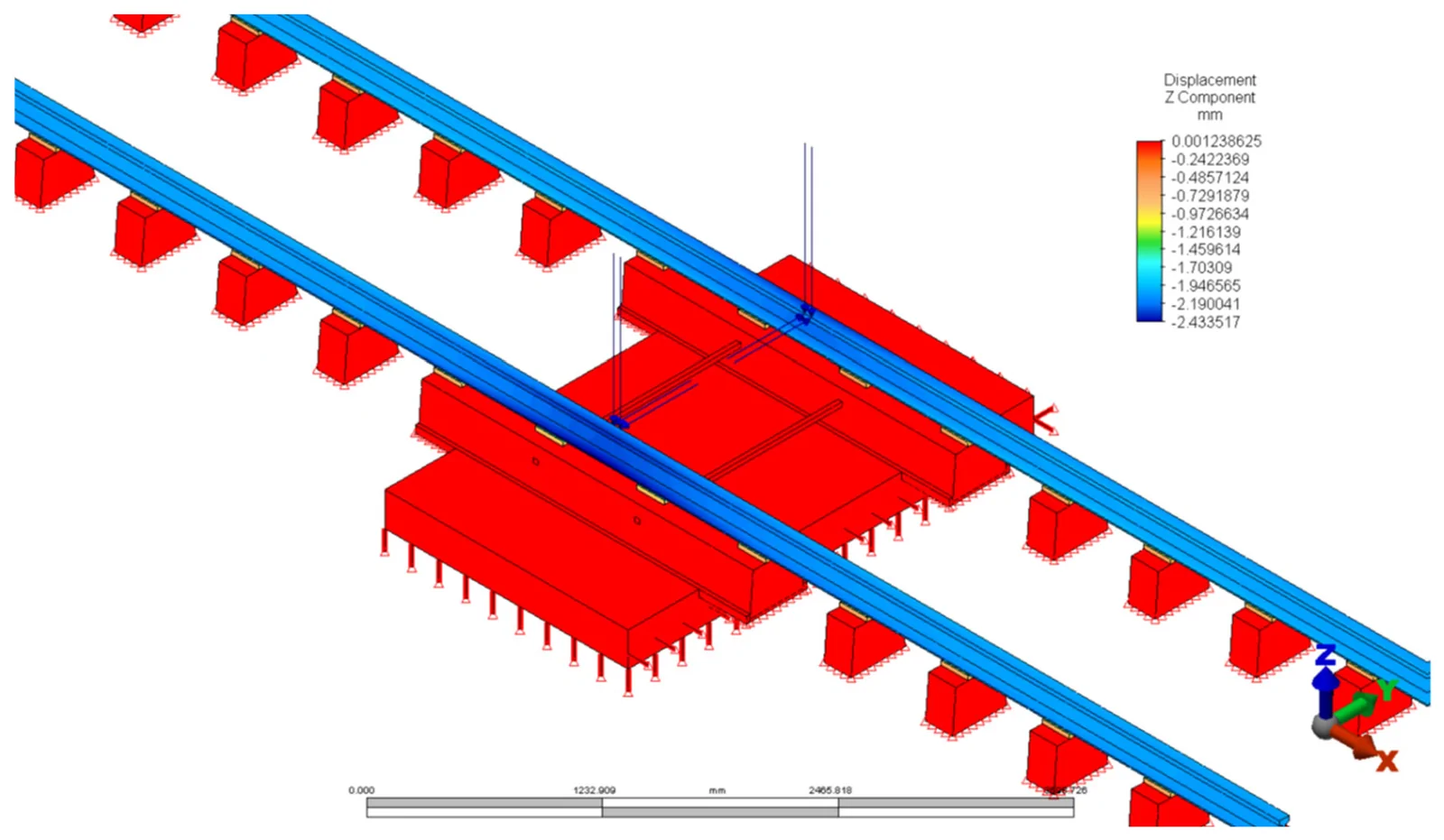
Vertical displacements of the LONGASF track model.

The calculated horizontal and vertical displacement distributions are presented in Figure 23 and Figure 24.

The maximum calculated horizontal and vertical displacements were 2.439 and 2.434 mm, respectively. Thus, for the conservative foundation modulus adopted in the extended model, the calculated displacements did not exceed approximately 2.5 mm. These results provide a preliminary assessment of the structural response of the LONGASF system; however, evaluation against operational acceptance criteria requires further model validation and field measurements.

The experimental tests were used to identify selected mechanical parameters of the fastening system, but they did not constitute validation of the complete LONGASF numerical model. In particular, the calculated rail, sleeper, and supporting-layer responses were not compared with measurements obtained from a full-scale asphalt-supported LONGASF test section. The numerical results should therefore be interpreted as unvalidated parameter studies showing the relative influence of the adopted stiffness and modelling assumptions. Quantitative prediction of the in-service response requires validation against full-scale measurements under moving railway vehicles.

## 8. Conclusions

This study combined previously unpublished experimental results for selected mechanical properties of the ICOSTRUN-03 fastening system with simplified dynamic and three-dimensional numerical analyses of the LONGASF ballastless track structure. After 2.3 million loading cycles, the longitudinal rail resistance remained above 12.6 kN, the resistance determined using the supplementary procedure decreased by approximately 6.8%, and the vertical fastening stiffness increased from 36.3 to 43.6 MN/m. These changes remained within the limits adopted for the experimental assessment. However, the repeatability measurements were obtained from a single specimen, and between-specimen variability could not be quantified.

The numerical analyses demonstrated that foundation stiffness strongly influenced the predicted rail and sleeper response. For the representative foundation modulus of $Cb=100\;MN/m^{3},$ the displacement criterion was satisfied throughout the investigated speed range, whereas the vibration-velocity and acceleration screening limits were exceeded at 250 km/h. The quasi-static analyses produced maximum vertical displacements of 1.323 and 1.246 mm and maximum transverse displacements of 2.587 and 1.571 mm for the investigated support-stiffness variants. In the extended three-dimensional model, the maximum calculated horizontal and vertical displacements were 2.439 and 2.434 mm, respectively.

Because the models were not validated at the complete track-system level and did not include coupled vehicle–track interaction, the results should be interpreted as a comparative parameter study rather than as confirmed predictions of operational performance. In particular, 250 km/h represents an investigated calculation case and not a validated permissible speed. Further research should focus on full-scale validation under moving vehicles, tests on independent fastening specimens, coupled vehicle–track modelling, and temperature- and frequency-dependent characterization of the asphalt layer.

## Figures and Tables

**Table 1:** Statistical summary of the preliminary repeated measurements performed on specimen LB/28/12-01.

Parameter	Number of Repeated Measurements	Individual Results	Mean	Standard Deviation	Coefficient of Variation	Range
Longitudinal rail resistance [kN]	4	12.6; 12.5; 12.6; 12.5	12.55	0.06	0.46%	12.5–12.6
Vertical stiffness [MN/m]	3	35.7; 35.7; 37.5	36.30	1.04	2.86%	35.7–37.5
Transverse rail resistance at 1 mm [kN]	5	30.7; 38.1; 36.7; 37.6; 39.6	36.54	3.43	9.39%	30.7–39.6

**Table 2:** Experimental results obtained for the ICOSTRUN-03 fastening system and the LONGASF track assembly.

Parameter	Initial Value	After 1,250,000 Cycles—Tangent Track	After 2,300,000 Cycles—Tangent Track and Curve
Longitudinal rail resistance according to PN-EN 13146-1:2003 [39] [kN]	>12.6	>12.6	>12.6
Longitudinal rail resistance determined using the special procedure [kN]	26.5	—	24.7
Vertical stiffness [MN/m]	36.3	39.2	43.6
Transverse rail resistance at 1 mm displacement [kN]	36.5	—	—

**Table 3:** Numerical parameters adopted in the simplified model of the LONGASF track system.

Parameter	Value
**Fastening stiffness**	
Transverse direction	*k*_r,y_ = 37.0 MN/m
Longitudinal direction	*k*_r,x_ = 46.8 MN/m
Vertical direction	*k*_r,z_ = 43.6 MN/m—value measured after 2,300,000 loading cycles
**Viscous damping coefficient**	
Transverse direction	*c*_r,y_ = 3600 Ns/m
Longitudinal direction	*c*_r,x_ = 2600 Ns/m
Vertical direction	*c*_r,z_ = 1600 Ns/m
**Other parameters**	
Longitudinal sleeper mass per rail and per unit length	866 kg/m
Effective distributed mass of the sleeper, associated asphalt, and protective ballast cover	1091 kg/m
Distributed vertical stiffness of the asphalt foundation	*U*_b,z_ = 67.5, 90, 135 and 180 MN/m^2^ (MN/m per metre length of the longitudinal sleeper)
Vertical damping of ballast cover and foundation	*c*_bj,z_ = 49,000 Ns/m
Distributed transverse stiffness of the supporting structure	*U*_p_ = 10 and 15 MN/m^2^ (MN/m per metre length of the longitudinal sleeper)
Damping of ballast cover and asphalt layer (small displacements)	*c*_bj,z_ = 5000 Ns/m^2^
Young’s modulus of concrete sleeper	*E*_b_ = 35,000 MPa
Poisson’s ratio of concrete sleeper	*ν* = 0.17
Cross-sectional area of sleeper	*A*_b_ = 0.1125 m^2^
Moment of inertia	*I*_z_ = 84,375 cm^4^ *I*_y_ = 126,633 cm^4^
Rail section	60E1
Train speed	0, 50, 100, 150, 200, 250 km/h
Reference vehicle	EP09 locomotive
Dynamic amplification factor (vertical loads)	ψ = 1 + (18.9 × 10^−6^)·v^2^ v-train speed expressed in km/h;
Lateral load	Y = 0.122·Q Q-vertical wheel load adopted in the simplified model.

## Data Availability

The original contributions presented in this study are included in the article. Further inquiries can be directed to the corresponding author.
